# All-Inorganic Hydrothermally Processed Semitransparent Sb_2_S_3_ Solar Cells with CuSCN as the Hole Transport Layer

**DOI:** 10.1021/acsaem.3c02492

**Published:** 2024-02-05

**Authors:** Pankaj Kumar, Martin Eriksson, Dzmitry S. Kharytonau, Shujie You, Marta Maria Natile, Alberto Vomiero

**Affiliations:** † Division of Materials Science, Department of Engineering Sciences and Mathematics, Luleå University of Technology, SE-971 87 Luleå, Sweden; ‡ Electrochemistry and Corrosion Laboratory, Jerzy Haber Institute of Catalysis and Surface Chemistry, Polish Academy of Sciences, 30-239 Krakow, Poland; § National Research Council (CNR), Institute of Condensed Matter Chemistry and Technologies for Energy (ICMATE), via F. Marzolo 1, 35131 Padova, Italy; ∥ Department of Chemical Sciences, University of Padova, via F. Marzolo 1, 35131 Padova, Italy; ⊥ Department of Molecular Sciences and Nanosystems, Ca’ Foscari University of Venice, via Torino 155, 30172 Venezia Mestre, Italy

**Keywords:** copper(I) thiocyanate, antimony sulfide solar cells, hydrothermal deposition, hole transport layer, semitransparent solar cells, average visible transmittance, thin film solar cells

## Abstract

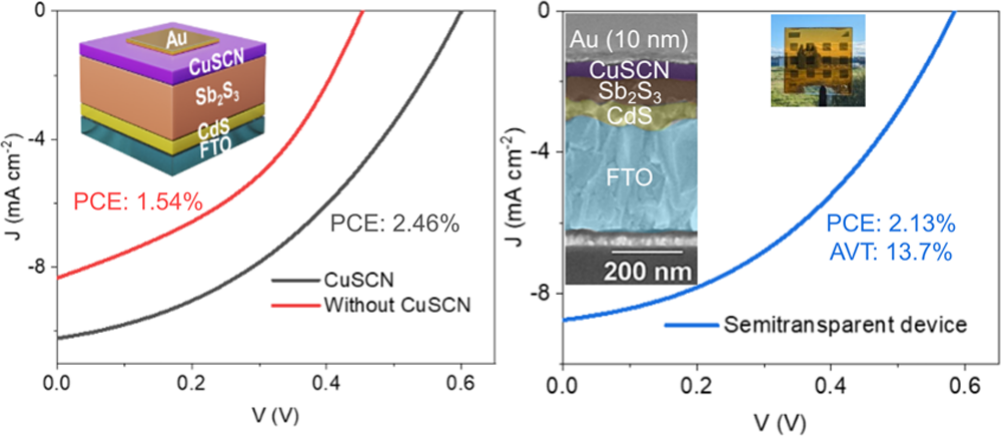

An inorganic wide-bandgap hole transport layer (HTL), copper(I) thiocyanate (CuSCN), is employed in inorganic planar hydrothermally deposited Sb_2_S_3_ solar cells. With excellent hole transport properties and uniform compact morphology, the solution-processed CuSCN layer suppresses the leakage current and improves charge selectivity in an n-i-p-type solar cell structure. The device without the HTL (FTO/CdS/Sb_2_S_3_/Au) delivers a modest power conversion efficiency (PCE) of 1.54%, which increases to 2.46% with the introduction of CuSCN (FTO/CdS/Sb_2_S_3_/CuSCN/Au). This PCE is a significant improvement compared with the previous reports of planar Sb_2_S_3_ solar cells employing CuSCN. CuSCN is therefore a promising alternative to expensive and inherently unstable organic HTLs. In addition, CuSCN makes an excellent optically transparent (with average transmittance >90% in the visible region) and shunt-blocking HTL layer in pinhole-prone ultrathin (<100 nm) semitransparent absorber layers grown by green and facile hydrothermal deposition. A semitransparent device is fabricated using an ultrathin Au layer (∼10 nm) with a PCE of 2.13% and an average visible transmittance of 13.7%.

## Introduction

Antimony sulfide (Sb_2_S_3_) is a promising emerging absorber layer for thin film photovoltaics because of its high absorption coefficient (1.8 × 10^5^ cm^–1^ at 450 nm), suitable optical bandgap [1.7 eV with theoretical Shockley–Queisser limit of power conversion efficiency (PCE) = 28.64%], long-term stability, elemental abundance, and low processing temperatures.^1,2^ The relatively high bandgap combined with a high absorption coefficient makes it a potential candidate for semitransparent solar cell applications.

Sb_2_S_3_ has a relatively low doping concentration and thus has a quasi-intrinsic nature. Doping Sb_2_S_3_ is difficult due to its ribbon-like crystal structure.^3^ An n-i-p-type structure, with an electron transport layer (ETL, n-type) and hole transport layer (HTL, p-type), can effectively relax the requirement on the doping density of the Sb_2_S_3_ absorber layer.^4^ The built-in electric field n-i-p solar cell is defined by the Fermi level difference between the ETL and HTL and charge extraction is expected to improve.^4^ The top-performing Sb_2_S_3_ solar cells in recent years employ an n-i-p planar device structure with maximum PCE exceeding 8%.^5,6^

An organic small molecule, 2,2′,7,7′-tetrakis(*N*,*N*-di-*p*-methoxyphenylamine)-9,9′-spiro-fluorene (Spiro-OMeTAD), or a polymer, poly(3-hexylthiophene-2,5-diyl) (P3HT), have been the materials of choice as HTL in the most efficient Sb_2_S_3_ solar cells.^5,7−9^ In general, these expensive organic HTLs suffer from inherent chemical and thermal instability. Therefore, considerable research efforts have been devoted to the search for suitable inorganic HTL materials.^10,11^ Among these inorganic HTLs, CuSCN is a cheaper, thermally stable alternative with sufficient hole mobility and transparency. Additionally, CuSCN has suitable properties to act as efficient HTL with a wide bandgap (*E*_g_) of ∼3.6 eV, suitable field-effect mobility values reported up to 0.4 cm^2^ V^–1^ s^–1^, and matched energy levels with Sb_2_S_3_ (valence band, *E*_v_ = −5.4 eV with respect to vacuum).^12−14^

The efficiencies remained lower than 2% for planar Sb_2_S_3_ solar cells with CuSCN as the HTL layer.^15^ In comparison, those using Spiro-OMeTAD as HTL have achieved a PCE of 8%.^5^ The major limitation in Sb_2_S_3_ solar cells comes from high voltage loss (*V*_loss_ = *E*_g_/e – *V*_OC_, where *E*_g_ is the bandgap of the light absorbing semiconductor, e is the elementary electronic charge, and *V*_OC_ is the open circuit voltage), which is more than 0.9 V for state-of-the-art Sb_2_S_3_ solar cells based on Spiro-OMeTAD.^5^ In comparison, the top-performing silicon and perovskite solar cells have achieved V_loss_ as low as 0.3–0.5 V.^16^ For CuSCN-based planar Sb_2_S_3_ devices, the *V*_OC_ values are even lower.^15^ Therefore, considering the promising physical and optoelectronic characteristics, CuSCN needs more investigation and optimization, especially targeting improvement in *V*_OC_.

In our previous work,^15^ CuSCN showed promising results in planar all-inorganic Sb_2_S_3_ solar cells where the absorber layer was deposited via chemical bath deposition (CBD). The devices with the structure FTO/TiO_2_/Sb_2_S_3_/CuSCN/Au achieved a PCE of 1.75%. One of the major limitations of the device’s performance was the low-quality nonuniform Sb_2_S_3_ film produced by CBD with oxygen impurities and unfavorable crystal orientations. Thus, the optimum thickness of Sb_2_S_3_ was limited to ∼50 nm with island-like morphology resulting in unavoidable HTL/ETL direct contacts.^15^ In recent years, planar Sb_2_S_3_ solar cells based on an alternative green and facile solution-based approach (the hydrothermal deposition method) have consistently achieved PCEs higher than 6% (albeit with Spiro-OMeTAD as HTL).^6,17,18^ In this work, to further improve the performance, a fully inorganic planar hydrothermally processed Sb_2_S_3_ solar cell based on CuSCN as the HTL is reported. A detailed optoelectronic characterization of CuSCN for HTL applications is followed by the simulation of solar cell devices with and without CuSCN. In the fabricated devices, the PCE of the solar cell with CuSCN HTL (device structure: FTO/CdS/Sb_2_S_3_/CuSCN/Au) was improved by up to 60% in comparison to the device without HTL. The obtained PCE of 2.46% is one of the highest among previously reported CuSCN-based planar Sb_2_S_3_ solar cells. Moreover, the inclusion of the CuSCN layer becomes even more consequential when the thickness of the Sb_2_S_3_ layer is reduced to below 100 nm. In these ultrathin Sb_2_S_3_ solar cells, the devices with the CuSCN HTL maintain high *V*_OC_ even at a thickness of 60 nm because of the shunt-blocking effect of the CdS/CuSCN heterojunction. On the other hand, the HTL-free devices are highly shunted for Sb_2_S_3_ thickness below 100 nm. These results suggest that CuSCN is a promising, cheaper, and stable alternative to expensive and inherently unstable organic HTLs. Furthermore, as demonstrated, the hydrothermal method can produce ultrathin Sb_2_S_3_ films for semitransparent solar cell applications. Thus, a proof-of-concept semitransparent device (with a 10 nm Au top electrode) is reported with a PCE of 2.13% and an average visible transmittance (AVT) of 13.7%.

## Experimental Section

Device fabrication: FTO substrates (TEC 15) were cleaned using Hellmanex III solution (2 vol % in distilled water), distilled water, acetone, and isopropanol, respectively, for 10 min each, followed by drying in an oven for 10 min at 120 °C. A CdS layer was deposited following a conventional CBD recipe.^19^ The deposition time was adjusted to achieve a thickness of 70–80 nm. Sb_2_S_3_ films were deposited using a hydrothermal deposition method. 0.02 M potassium antimonyl tartrate trihydrate (≥99%, VWR chemicals) and 0.08 M sodium thiosulfate pentahydrate (>99.5%, VWR chemicals) were added to a 30 mL Teflon lined autoclave. The hydrothermal reaction was carried out for varying times (60–240 min) at 135 °C according to ref (18). The coated films were rinsed in distilled water and annealed in a tube furnace in argon at 350 °C (ramp rate 10 °C per min) for 10 min.^18^ CuSCN was spin-coated from a diethyl sulfide at 4000 rotations per min (rpm) and dried at 90 °C for 10 min, following our previous report.^15^ Au top contact was sputtered using a compact sputter coater (Leica EM ACE200) and the device area was defined by a laser-cut metal mask with an area of 9 mm^2^. For a space charge limited current (SCLC) device, NiO_*x*_ hole injection contact was prepared via spin coating following previously reported recipes.^20,21^ Briefly, 276.3 mg of Ni(NO_3_)_2_·6H_2_O was dissolved in 2-methoxyethanol (10 mL). After the mixture was stirred at 50 °C for 1 h, 100 μL of acetylacetone was added to the solution. After stirring for 1 h, the solution was filtered through a nylon filter (0.45 μm) and spin-coated on FTO at 3000 rpm and further annealed in air at 150 °C for 1 h. Control devices using an organic HTL P3HT (Ossila, M108) were prepared using the following conditions: a concentration of 10 mg mL^–1^ in chlorobenzene, spin-coated at 4000 rpm, and annealed in a tube furnace in an argon atmosphere at 120 °C for 10 min. To improve the contact with Au, a diluted (1:6 volume ratio in isopropanol) PEDOT:PSS (Ossila PH1000) was subsequently spin-coated on PEDOT:PSS at 2000 rpm and annealed in a tube furnace in the argon atmosphere at 120 °C for 10 min.^9,22^

### Characterization and Measurements

#### Optical Characterization

Transmittance/absorbance characterizations were performed by a Cary 5000 spectrophotometer. The optical bandgap, *E*_g,_ of thin films, was extracted using Tauc’s formula: (α*h*ν)^γ^ = *C* (*h*ν – *E*_g_), where α is the absorption coefficient, *h*ν is the photon energy, *C* is a constant, and γ takes the values of 2 or 1/2 for direct and indirect bandgap, respectively.^23^ The absorption coefficient, α was calculated using the equation: α = *A*ln10/*t*, where *A* is the absorbance and *t* is the thickness of the film (determined by cross-section scanning electron microscopy (SEM)).^24^ For both Sb_2_S_3_ and CuSCN films, both direct (γ = 2) and indirect (γ = 1/2) bandgaps were extracted from the Tauc plots. Both direct and indirect bandgaps have been calculated based on the previous reports for CuSCN^25^ and Sb_2_S_3_^26^ thin films. AVT was calculated using the equation:^27^
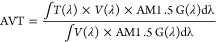
where λ is the wavelength, *T* (λ) is the transmission spectrum with air as a reference, *V* (λ) is the photopic response of the human eye, and AM1.5G represents the standard solar photon flux.

#### SCLC Mobility Characterization

In hole-only SCLC devices, the current varies linearly at low voltages, followed by a SCLC regime (assuming a trap-free semiconductor film) where the current is proportional to *V*^2^. In this SCLC regime, the Mott–Gurney equation^28^ was used to calculate the hole mobility, μ_h_, given by
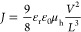
where ε_0_ is the permittivity of free space, ε_r_ = 5.1,^29^ which is the dielectric constant of CuSCN, *V* is the voltage, and *L* is the thickness of the film (∼100 nm). The thickness of CuSCN can be tuned simply by changing the spin-coating speed (Figure S1).

#### SEM Morphology and Cross-Section

Morphology and cross sections of films and devices were characterized by Magellan 400 Field Emission Scanning Electron Microscopy.

X-ray diffraction patterns (XRD) were collected on a PANalytical Empyrean X-ray diffractometer with a Cu K_α_ source.

#### Atomic Force Microscopy (AFM) and Kelvin Probe Force Microscopy (KPFM)

The AFM topography maps and KPFM contact potential difference (CPD) maps were recorded using an NTEGRA system from NT-MDT Spectrum Instruments. High-quality topography maps were recorded in semicontact mode using cantilever A on a HA_NC tip from NT-MDT with a nominal force constant of 12 N/m. The CPD was recorded with 2-pass KPFM in ambient conditions using an ElectriTap300-G tip from BudgetSensors having a nominal force constant of 40 N/m. For the thin films on FTO, conductive HQ:NSC18/Pt probes from μmasch with a nominal force constant of 2.8 N/m were used. The freshly sputtered Pt and Au samples were used as potential references and examined before and after every measurement. During the second pass, the cantilever was lifted 100 nm above the sample surface, and the amplitude of the applied AC voltage was 3 V. The sample substrate was connected to the ground during the entire measurement.

#### X-ray Photoelectron Spectroscopy (XPS)

XPS analysis was performed with a ThermoFisher Escalab QXi spectrometer using monochromatic Al Kα radiation and argon-assisted charge compensation. All signal positions are given in terms of binding energy. Both extended (survey: pass energy of 100 eV; dwell time of 20 ms; step of 1 eV) and detailed spectra (pass energy of 25 eV; dwell time of 50 ms; step of 0.1 eV) were collected. Spectra were analyzed with ThermoFisher Advantage software (2023 version). All quantifications were performed from detailed spectra.

#### Device Characterizations

The current density–voltage (*J*–*V*) measurements were performed using a Keithley 2400 source meter under simulated AM 1.5G irradiation (100 mW cm^–2^) with a standard 100 W Xe lamp-based solar simulator Oriel LCS-100 (default scan parameters: forward scan from −0.2 to 0.8 V; scan rate: 100 mV/s). The illumination intensity was calibrated by a monocrystalline silicon reference cell (Oriel 91150) P/N). The external quantum efficiency (EQE) was measured using the Rera SpeQuest quantum efficiency system, calibrated by silicon solar cell reference. Electrical impedance spectroscopy (EIS) measurements were performed using an electrochemical workstation (Solartron Analytical, ModulabXM) in the frequency range from 1 MHz to 1 Hz with an applied potential of 0.6 V in the dark. Equivalent circuit fitting was performed using an EIS Spectrum Analyzer developed by Bondarenko et al.^30^

#### SCAPS Simulation

A numerical simulation program, SCAPS-1D, developed by Burgelman et al., was used to simulate the device structure.^31^ The simulated structures consist of FTO/CdS/Sb_2_S_3_/CuSCN or P3HT/Au or HTL-free FTO/CdS/Sb_2_S_3_/Au. Equations governing the simulation software are described in Supplementary Note 1. The parameters and material properties are listed in Table S1, along with the corresponding references. The comparison with the reported literature is given in Table S2. The output of the simulations (along with nonideal conditions as discussed in Supplementary Note 2) are shown in Table S3 and Figure S2.

## Results and Discussion

### Material Characterization

**Figure 1:**
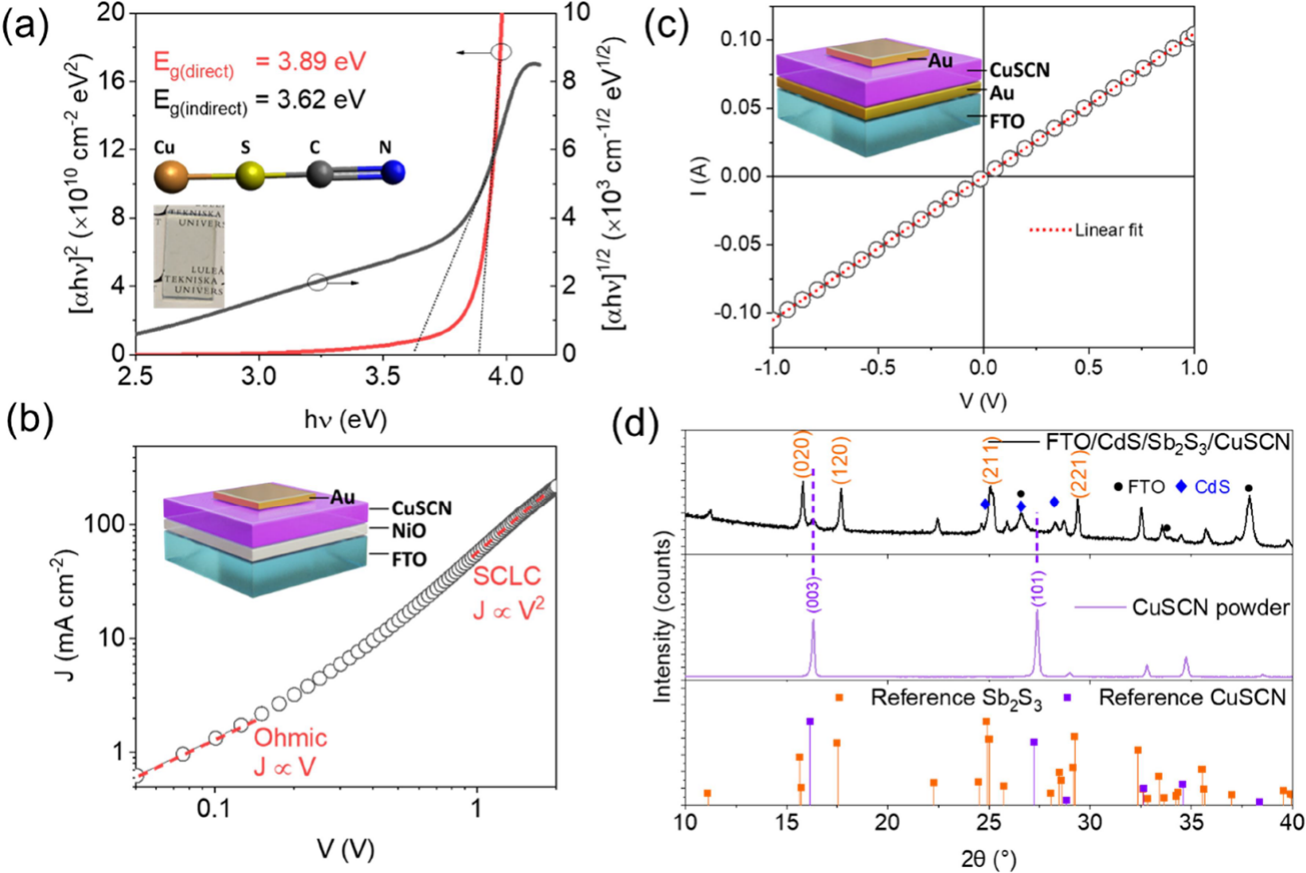
(a) Plots of (α*h*ν)^1/2^ and (α*h*ν)^2^ calculated for the CuSCN film. The inset shows a 60 nm CuSCN film coated on an FTO substrate. (b) *J–V* curves for SCLC devices showing the ohmic and SCLC region. The device structure is also shown. (c) *I*–*V* curve for conductivity measurement along with the device structure. (d) X-ray diffraction pattern of commercial CuSCN powder and the annealed CuSCN film on the Sb_2_S_3_ film. The reference patterns are also plotted for peak matching.

The absorption spectra of CuSCN and Sb_2_S_3_ films are shown in Figure S3a. The CuSCN layer is quite transparent (average transmittance >90% in the 300–800 nm range with an AVT of 93.5%) in the absorption region of Sb_2_S_3_ (Figure S3b), which is quite attractive for semitransparent and tandem Sb_2_S_3_ solar cells.^32^ Since there is no consensus in the literature about the type of bandgap (direct or indirect) for CuSCN,^33^ Tauc plots were used to extract both direct (*E*_g-direct_) and indirect (*E*_g-indirect_) optical bandgaps (Figure 1a). The CuSCN film showed a good straight-line fit for both direct and indirect types of optical transitions. The calculated *E*_g-direct_ and *E*_g-indirect_ were 3.89 and 3.62 eV, respectively, close to those reported elsewhere.^12,34^ The presence of an indirect *E*_g_ in CuSCN thin films has been linked to the observation of a long tail region in the absorption spectrum.^25^ Similarly, the direct and indirect optical bandgaps of Sb_2_S_3_ were ca. 1.76 and 1.58 eV, respectively, as reported elsewhere as direct^35^ or both direct and indirect^26,36^ (Figure S3c). Moreover, both direct and indirect bandgaps for Sb_2_S_3_ were predicted in DFT simulations.^26^ For thin film solar absorber applications, a direct bandgap type material is preferred because of its more efficient absorption around the bandgap.^37^ To evaluate the hole transport properties of the CuSCN films, hole-only mobility was calculated in a SCLC device with a device configuration (FTO/NiO/CuSCN/Au, Figure 1b). Both FTO/NiO and Au should make ohmic contact with CuSCN to ensure efficient hole injection.^38^ Hence, KPFM was used to determine the work function (WF) of these films.^39^ The morphology and CPD of CuSCN (70 and 200 nm) and NiO films on FTO are shown in Figure S4a–f. The measured work functions of these films (NiO: −5.09 eV, CuSCN-200 nm: −5.27 eV, and CuSCN-70 nm: −5.32 eV) are close to that of Au (taken as the reference with the WF of −5.1 eV) and confirm the p-type character of these films (NiO valence band: –5.4 eV).^21^ Notably, the work functions of CuSCN films were weakly dependent on the thickness or roughness of the films, which would ensure spatially uniform hole conduction properties (Figure S4g). The cross-sectional image of the SCLC device is also shown in Figure S4h. The hole mobility (μ_h_) was calculated in the SCLC regime as 1.74 (±0.61) × 10^–4^ cm^2^ V^–1^ s^–1^. This value is lower than those reported by other measurement techniques such as field-effect mobility (0.1 cm^2^ V^–1^s^–1^)^12^ or time-of-flight (∼10^–3^ cm^2^ V^–1^s^–1^).^40^ However, SCLC mobility is more realistic for solar cell application since the film thickness is the same range as the solar cell (the time-of-flight technique uses films in μm thickness range) and the mobility is calculated across the thickness of the film (in contrast to field-effect mobility, where mobility is calculated in in-plane direction).^40^ To measure the conductivity and verify the nature of CuSCN/Au contact (ohmic or Schottky), a device with the structure FTO/Au/CuSCN/Au was prepared (Figure 1c). An ohmic contact was formed between CuSCN and Au, which is important for efficient charge extraction and reduction of the recombination losses. The conductivity (σ) was calculated as 1.1 (±0.02) × 10^–5^ Ω^–1^ cm^–1^ from the ohmic slope of the *I*–*V* plot, *I* = σ(*A*/*d*)*V*,^41^ where *A* is the device area (0.09 cm^2^) and *d* is the thickness of the CuSCN layer measured using SEM cross-section images (Figure S4h). Using the mobility and conductivity values, a carrier concentration of 3.95 (±0.12) × 10^17^ cm^–3^ is calculated using the equation σ = *N*_A_eμ_h_, where *N*_A_ is the carrier density of acceptors in CuSCN, e is the elementary charge, and σ is the conductivity of the film. This is consistent with the values reported elsewhere.^29,42^

The XRD from the FTO/CdS/Sb_2_S_3_/CuSCN stack is studied and reported in Figure 1d. In the middle panel in Figure 1d, the diffraction patterns from the commercial CuSCN powder match well with reference JCPDS #29-0581, which confirms its β-phase structure. Furthermore, the weak peak at 16.1° in Figure 1d top panel is assigned to the (003) diffraction from the CuSCN film (60–70 nm) coated on Sb_2_S_3_. The strongest diffraction peaks below 35° are assigned to the orthorhombic Sb_2_S_3_ (JCPDS #42-1393, space group Pbnm).^36^ [*hk*1]-oriented crystal is known to have relatively rapid transport of photogenerated carriers and higher performance compared to [*hk*0]-orientation.^43^ The presence of [*hk*1]-oriented peaks such as (221) and (211) along with [*hk*0]-oriented peaks such as (120) and (020) suggest that there was no preferential orientation in the hydrothermally deposited Sb_2_S_3_ films.

### Device Simulation

**Figure 2:**
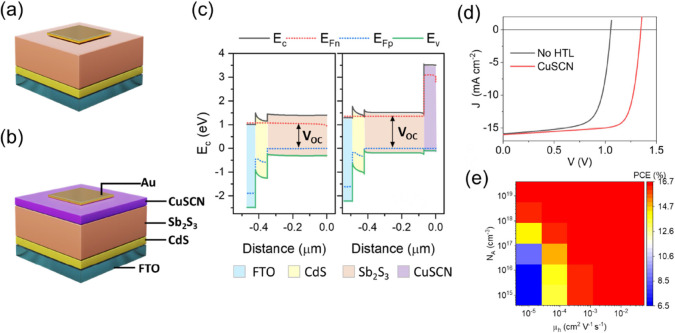
Device architecture of the planar Sb_2_S_3_ solar cell (a) without and (b) with CuSCN HTL. (c) Energy band diagrams of CdS/Sb_2_S_3_ and CdS/Sb_2_S_3_/CuSCN devices under AM1.5G irradiation at open circuit conditions. (d) Simulated *J–V* curves of the two cases: with and without CuSCN layer. (e) Contour plot for variation of PCE of the devices with acceptor density (*N*_A_) and hole mobility (μ_h_) of CuSCN HTL.

Although CuSCN has been successfully applied in perovskite solar cells, the reports of its application in planar Sb_2_S_3_ solar cells are limited.^44,45^ Moreover, there are several reports of high-performance Sb_2_S_3_ solar cells without the HTL layer (“HTL-free”).^46,47^ Therefore, drift-diffusion simulations for solar cell devices with and without CuSCN as the HTL were performed to study the theoretical influence of the addition of an HTL layer on the device performance in an n-i-p configuration. Figure 2a,b show the device structures without and with the HTL layer. Figure 2c shows the energy level diagrams for these devices under open circuit conditions under 1 sun illumination. The corresponding simulated *J–V* curves are shown in Figure 2d, with the material parameters fixed as in Table S1. The device without an HTL suffers from low *V*_OC_ and FF but comparable *J*_SC_ values (with CuSCN: PCE = 16.48%, *V*_OC_ = 1.35 V, *J*_SC_ = 16.04 mA cm^–2^, FF = 76.20%; Without HTL: PCE = 11.75%, *V*_OC_ = 1.05 V, *J*_SC_ = 15.91 mA cm^–2^, and FF = 70.08%). The addition of CuSCN with a higher conduction band (*E*_c_) (than that of Sb_2_S_3_ as represented in Figure 2c) suppresses the electron back transfer from Sb_2_S_3_ to top contact (Au) and reduces the charge recombination, thus increasing the FF. The theoretical maximum *V*_OC_ is defined as the quasi-Fermi-level splitting (QFLS) (inside the absorber layer) between the electron and hole Fermi levels (*E*_Fn_ – *E*_Fp_, represented by black and blue dotted lines in Figure 2c).^48^ Introducing CuSCN increases the selectivity of electrons and holes and thus reduces the level of recombination near the hole contact. On the other hand, without the CuSCN HTL, the quasi-Fermi level of electrons is further reduced near the top contact (here Au with work function (WF = −5.1 eV) was used). Thus, the net effect of CuSCN is an increase in the QFLS, thus increasing the *V*_OC_.^48^

However, since CuSCN will also add additional resistance, conductivity, which is a product of mobility and acceptor carrier concentration (doping density, *N*_A_) becomes an important parameter. Thus, the hole mobility (μ_h_) and acceptor carrier density (*N*_A_) are two of the most important parameters that influence the effectiveness of the HTL. It is also possible to tune these properties using doping or other post-treatments of CuSCN.^14,49^ A contour plot showing the effect of varying these two parameters (other parameters are the same as Table S1) is plotted in Figure 2e. The PCE of devices based on the CuSCN HTL layer is highly sensitive at lower values of both μ_h_ and *N*_A_ and saturates beyond certain minimum values (μ_h_ > 10^–4^ cm^2^ V^–1^s^–1^ and *N*_A_ > 10^17^ cm^–3^). These values are quite achievable in CuSCN thin film without doping as in the current investigation and reported elsewhere.^29,50^ Furthermore, a comparison table of reported simulations of planar Sb_2_S_3_ solar with different device structures is presented in Table S2. The device with a CuSCN HTL in the ideal case (low bulk defect density of 10^12^ cm^–3^ and no interface traps) should theoretically perform as well as other HTLs. Some selected experimental reports are also listed. *V*_OC_ loss is the major loss factor in the experimental devices compared with the simulations.

### Photovoltaic Performance

**Figure 3:**
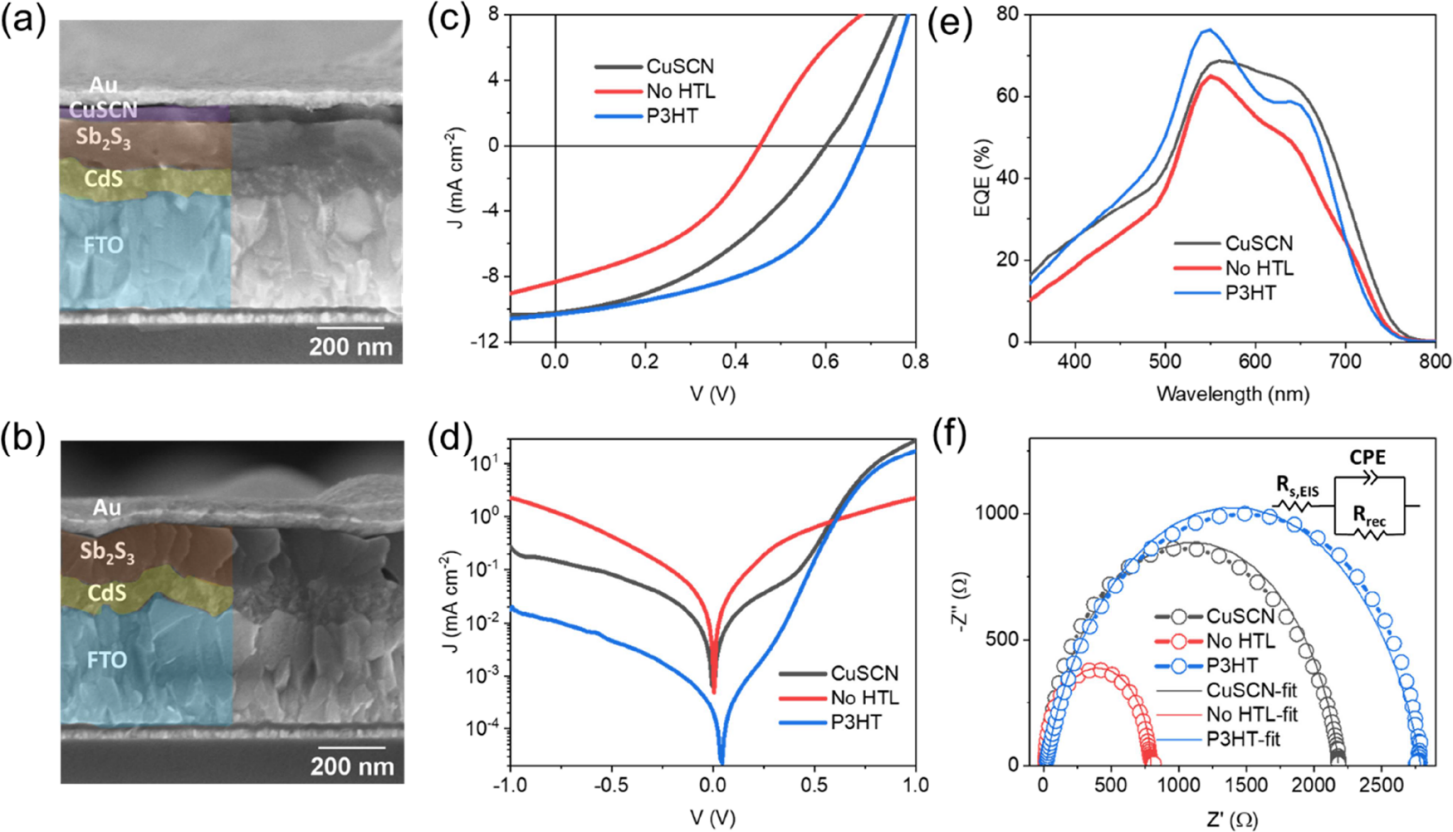
Cross-sectional SEM image of a typical solar cell (a) with and (b) without a CuSCN HTL. (c) Light *J–V* curves, (d) dark *J–V* curves, (e) external quantum efficiency of solar cells, and (f) Nyquist plots for devices with CuSCN and P3HT HTLs and without HTL.

The cross-sectional SEM images of the typical devices with and without CuSCN HTL are shown in Figure 3a,b, respectively. The compact CuSCN film is in intimate contact with the Sb_2_S_3_ layer. The top surface SEM and AFM images of Sb_2_S_3_ before and after CuSCN coating are shown in Figure S5a–d_**.**_ CuSCN film showed full coverage of Sb_2_S_3_ film, filling the pinholes in the films. With conformal and compact coverage, CuSCN would reduce the shunt paths and leakage currents in the solar cells.^51^ Notably, the pinholes (morphology) were not taken into consideration in the SCAPS simulated devices but would determine the property of physical contact with the top electrode, especially for the solution-processed Sb_2_S_3_ layer with noncompact morphology with pinholes.^52^

Figure 3c shows the *J*–*V* curves of champion Sb_2_S_3_ solar cells with and without the CuSCN HTL layer along with P3HT HTL as a control reference device.^53^ The corresponding photovoltaic performance parameters are listed in Table 1, and the thicknesses of Sb_2_S_3_ and HTLs are given in the footnote. The statistical distribution of these solar cells is shown in Figure S6**.** Higher reproducibility is clearly an added advantage of adding CuSCN (the mean and standard deviation of all the parameters are listed in Table 1). The thickness of CuSCN was optimized by varying the spin-coating RPMs (Figure S1a–c), and the corresponding device performance parameters are listed in Table S4. The champion device with CuSCN obtained a PCE of 2.46% (*V*_OC_ = 0.60 V, *J*_SC_ = 10.22 mA cm^–2^, and FF = 40.13%). A typical forward and reverse scan showed less than 5% hysteresis (Figure S7), suggesting efficient charge transfer across the device. The champion device without an HTL showed a PCE of 1.54% (*V*_OC_ = 0.45 V, *J*_SC_ = 8.36 mA cm^–2^, and FF = 40.63%). The reference device with P3HT showed both higher *V*_OC_ and FF, resulting in a PCE of 3.42%. Sb_2_S_3_ and P3HT have been reported to form an intimate chelating bonding contact and it possibly passivates the interface.^54^

**Table 1:** Device Performance Parameters of Solar Cell Devices With and Without a CuSCN HTL

		*V*_OC_ (V)	*J*_SC_ (mA cm^–2^)	FF (%)	PCE (%)	*R*_s_ (Ω cm^2^)	*R*_sh_ (Ω cm^2^)
CuSCN	best	0.60	10.22	40.13	2.46	24.5	347.2
	mean ± SD^a^	0.62 ± 0.02	8.86 ± 0.85	41.9 ± 1.89	2.30 ± 0.09	27.4 ± 2.7	340.3 ± 38.5
no HTL	best	0.45	8.36	40.63	1.54	21.7	134.9
	mean ± SD^a^	0.42 ± 0.03	8.30 ± 0.22	38.40 ± 1.84	1.35 ± 0.18	23.7 ± 1.4	118.9 ± 14.3
P3HT	best	0.68	10.34	48.41	3.42	15.2	317.5
	mean ± SD^a^	0.68 ± 0.05	10.07 ± 0.27	48.24 ± 0.40	3.31 ± 0.10	15.0 ± 0.5	317.3 ± 16.1

a Mean and standard deviation (SD) calculated from the average of the top 6 devices for CuSCN and P3HT-based devices and 5 devices for no-HTL devices. The thicknesses of the absorber layers are as follows: 200 nm for CuSCN-based devices, 260 nm for no-HTL devices, and 110–120 nm for P3HT devices. CuSCN layer was 60–70 nm thick and P3HT (with PEDOT:PSS modification layer) was about 30–40 nm.

The efficiencies of these HTL-free devices are lower compared to the literature with similar device structures (FTO/CdS/Sb_2_S_3_/Au, with reported PCEs over 5%) which points to the room for potential improvements in the PCEs via optimization of ETL and Sb_2_S_3_ processing conditions.^46,47^ With the aim of shedding light on the possible causes of the lower performances of these devices with respect to most planar Sb_2_S_3_ solar cells with similar structures reported in the literature, X-ray photoelectron spectroscopic analysis was carried out on the FTO/CdS/Sb_2_S_3_. Besides confirming the growth of Sb_2_S_3_, the XPS analysis also revealed a significant presence of Sb_2_O_3_ on the surface (Figure S8 and Supplementary Note 3), which is detrimental to the device performance as reported elsewhere.^36,55^ Choi et al.^56^ reported a high concentration of deep defects (up to 5 × 10^14^ cm^–3^ at 0.52 eV above the valence band) due to oxide impurities. Furthermore, low performances can be attributed to the discontinuous morphology with pinholes and smaller grains (<500 nm) compared to dense morphology in the reported literature for hydrothermally deposited Sb_2_S_3_ films.^57^ Moreover, as shown in the energy-dispersive X-ray spectroscopy (EDS) measurements (Figure S9), the atomic ratio of S/Sb for Sb_2_S_3_ films on FTO is 1.45, suggesting that the Sb_2_S_3_ film is S-deficient. Sulfur vacancy (*V*_s_) also contributes to deep traps and associated recombination losses.^57^

Consequently, a huge discrepancy exists between the simulated and experimental data. To make the simulations more realistic and to understand the effects of nonidealities, three nonideal cases were added to the device simulation one by one. The “ideal” case represents the baseline simulation in Figure 2d. In the subsequent simulations, actual device parameters and deep and interface defects were introduced (Supplementary Note 2). The effects of these nonidealities can be seen in calculated *J*–*V* curves (Figure S2) and Table S3. The results are consistent with our conclusion that the HTL layer plays a significant role in improving the performance and limiting the shunts. The limitation of these 1-D simulations in replicating the actual devices is also discussed briefly in Supplementary Note 2.

The rollover effect (S-shape in the *J–V* curve Figure 3c) can be seen in the device without the HTL layer, which is characteristic of the top contact barrier (nonohmic contact).^58^ Without the HTL layer, the Sb_2_S_3_ interface would form a Schottky contact with Au, which will act in a reverse direction to the main junction (CdS/Sb_2_S_3_), reducing the *V*_OC_. This observation is well established and a selenization treatment would often solve the nonohmic Sb_2_S_3_/Au contact, by lowering the *E*_g_ (Sb_2_Se_3_ formation) at the HTL interface.^52,59^ Series and shunt resistances of the two devices in light were calculated from the linear slopes near open circuit (*1/R*_s_ = d*J*/d*V*|_*V*=Voc_) and short circuit (*1/R*_sh_ = d*J*/d*V*|_*V*=0 V_) conditions, respectively.^60^ Average *R*_s_ of the devices with no HTL was slightly lower than that of the device with CuSCN (23.7 ± 1.4 Ω cm^2^ for no HTL vs 27.4 ± 2.7 Ω cm^2^ for CuSCN-based devices). However, the shunt resistance for the CuSCN device is much higher (340.3 ± 38.5 Ω cm^2^) compared to that of the device without the HTL layer (118.9 ± 14.3 Ω cm^2^). Higher shunt resistance achieved by employing the CuSCN HTL layer means reduced leakage currents and thus a higher average FF and *V*_OC_. P3HT-based devices showed both lower *R*_s_ and higher *R*_sh_. Serious leakage currents and a lack of selectivity (low rectification ratio) can be seen in the dark *J*–*V* curves without HTL along with a lack of diode rectification characteristics (Figure 3d). The dark reverse current is significantly reduced signifying the role of HTL (CuSCN or P3HT) in improving the charge selectivity. The shallow conduction band of both the HTLs (−1.5 eV for CuSCN and −3.2 eV for P3HT)^36^ reduces the electron injection from Au to Sb_2_S_3_ and vice versa.^61^

The short circuit current density, *J*_SC,_ is influenced by the absorption, charge separation, and collection properties of the device in the absence of an externally applied field. To study the wavelength-dependent charge collection efficiency of the solar cells, EQE measurements were carried out (Figure 3e). The integrated J_SC,EQE_ values were close to those measured via the solar simulator (9.00 mA cm^–2^ for no-HTL device, 11.07 mA cm^–2^ for the CuSCN device, and 10.81 mA cm^–2^ for the P3HT device). A relative improvement in EQE in CuSCN and P3HT-based devices in higher wavelength regions (>600 nm) can be attributed to lower shunt losses (as seen in low leakage currents in dark *J–V* curves in Figure 3d) along with better hole collection (electron reflection) efficiency of the hole contact since the electron–hole pairs are created near the hole contact for long-wavelength photons and electrons can easily recombine with Au contact in absence of the electron blocking HTL. In the low wavelength regions (<500 nm), the improvement in EQE of both HTL-based devices can be attributed to efficient hole extraction. In this low wavelength region, photons are completely absorbed near the CdS/Sb_2_S_3_ interface. The generated electrons and holes formed near the electron contact are separated by the built-in electric field and collected by CdS and hole contact, respectively. The built-in potentials can be estimated from crossover point of light and dark *J–V* curves as shown in Figure S10.^62^ Electrons can be easily collected by CdS since they are created near the CdS layer, while the holes need to travel through the low Sb_2_S_3_ absorber layer to the hole contact (Au or HTL/Au). Introduction of an HTL reduces the Schottky barrier (evidenced by S-shaped rollover in *J–V* in Figure 3c), increases built-in potential (0.46, 0.66, and 0.75 V, respectively for no HTL, CuSCN, and P3HT-based devices as shown in Figure S10), reduces the shunt losses (lower leakage currents in dark *J*–*V* in Figure 3d), and possibly improves the hole extraction rate due to favorable band alignment.^51,63^

Furthermore, charge transport and recombination characteristics of the cells with and without CuSCN HTL were studied by using electrical impedance spectroscopy measurements (EIS) in the dark (Figure 3f). The Nyquist plot showed a single depressed semicircle, which is modeled by a series resistance component, *R*_S,EIS_ (combining all resistances including that of electrodes) in series with a parallel combination of recombination resistance (*R*_rec_)—constant phase element, CPE. *R*_s,EIS_ value for the device in the dark without HTL (12.4 vs 20.0 Ω) is lower than that with CuSCN, while the *R*_rec_ for the CuSCN device is substantially higher. With CuSCN HTL, *R*_rec_ has increased (from 780 to 2161 Ω), implying reduced recombination in the device and, thus higher *V*_OC_. P3HT device showed further improved recombination resistance (2750 Ω) possibly due to its passivating contact with P3HT.^9,54^ Since the n-sides of the devices (CdS/Sb_2_S_3_) and WFs of electrodes are identical in all cases, these*R*_rec_ values are primarily associated with the hole contact properties.

### Surface Potential

**Figure 4:**
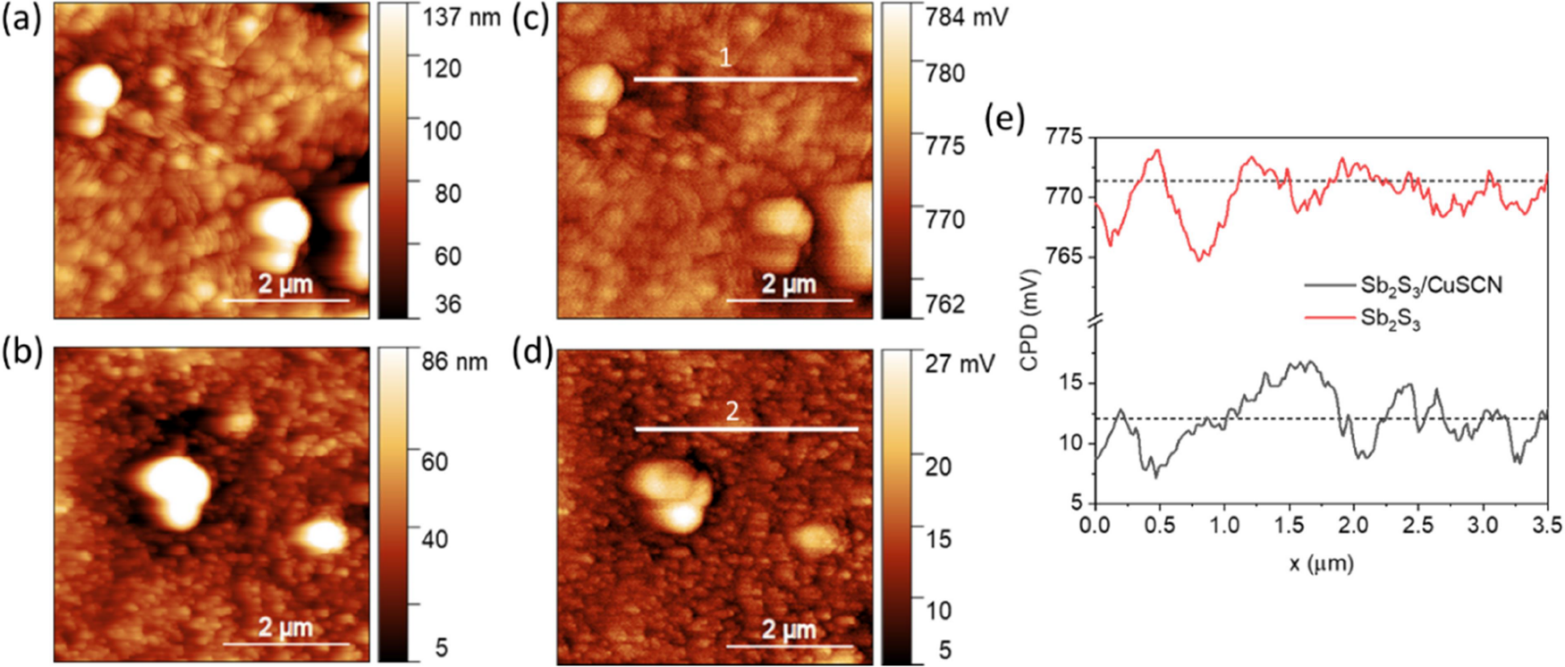
AFM topography maps of (a) hydrothermally deposited Sb_2_S_3_ and (b) CuSCN coated on the Sb_2_S_3_ film and their corresponding KPFM maps: (c) Sb_2_S_3_ and (d) Sb_2_S_3_/CuSCN film. (e) Line profile of the surface potential of (c) and (d) along lines 1 and 2, shown inside the CPD scans, respectively.

To further investigate the improvement of device performance (especially the *V*_OC_), surface potentials before and after CuSCN deposition on Sb_2_S_3_ were mapped using KPFM. The variations in CPD between the tip and the sample, across the entire scan area (including grain boundaries and morphology as shown in Figure 4a,b) are below 10 mV for both films (Figure 4c,d). This fluctuation is lower than the thermal energy per electron (*kT*/e = 25.7 mV, where *k* is the Boltzmann constant, *T* is temperature, and e is the elementary charge) (Figure 4e). This indicates that the CuSCN was deposited conformally on the Sb_2_S_3_ (covering even the subμm sized hemispherical grains of Sb_2_S_3_) and the electrical properties do not vary across the film. The CPD of CuSCN film on Sb_2_S_3_ (12.1 ± 0.7 mV) translates to an average WF value (*E*_F_) of −5.08 eV (calibrated against the Au electrode with a WF of −5.10 eV). The deviation of the WF of CuSCN films on Sb_2_S_3_ (∼0.2 eV) from those on the FTO substrates (Figure S4g) can be attributed to slightly different laboratory conditions (temperature and humidity), which might affect the measurement. The measured values for *E*_F_ of CuSCN are in close agreement with previous reports.^12,64^ Similarly, the work function of Sb_2_S_3_ (−4.32 eV) is close to the middle of the bandgap (considering *E*_v_ = −5.72 eV,^65^ and *E*_g_ = 1.76 eV), suggesting a weak n-type character. The obtained work function value is close to the previously reported value.^66^ Moreover, the measured WF values CuSCN films (−5.08 to −5.32 eV) are close to the valence band (*E*_v_ = −5.4 eV),^12^ suggesting a p-type character with high carrier density (>10^17^ cm^–3^).^50^

Another important attribute of employing CuSCN with a high *E*_F_ (and *E*_v_) is the elimination of the Fermi-level pinning phenomenon. Savadogo and Mandal^67^ reported that the Schottky barrier height was independent of the work function of the top contact and concluded the Fermi level was pinned near the midgap of Sb_2_S_3_ (around −4.5 eV). Hence, isolating the Sb_2_S_3_ layer from top contact via a CuSCN HTL eliminates a major *V*_OC_-limiting phenomenon (Fermi-level pinning) because of its high *E*_F_.

### Semitransparent Solar Cells

**Figure 5:**
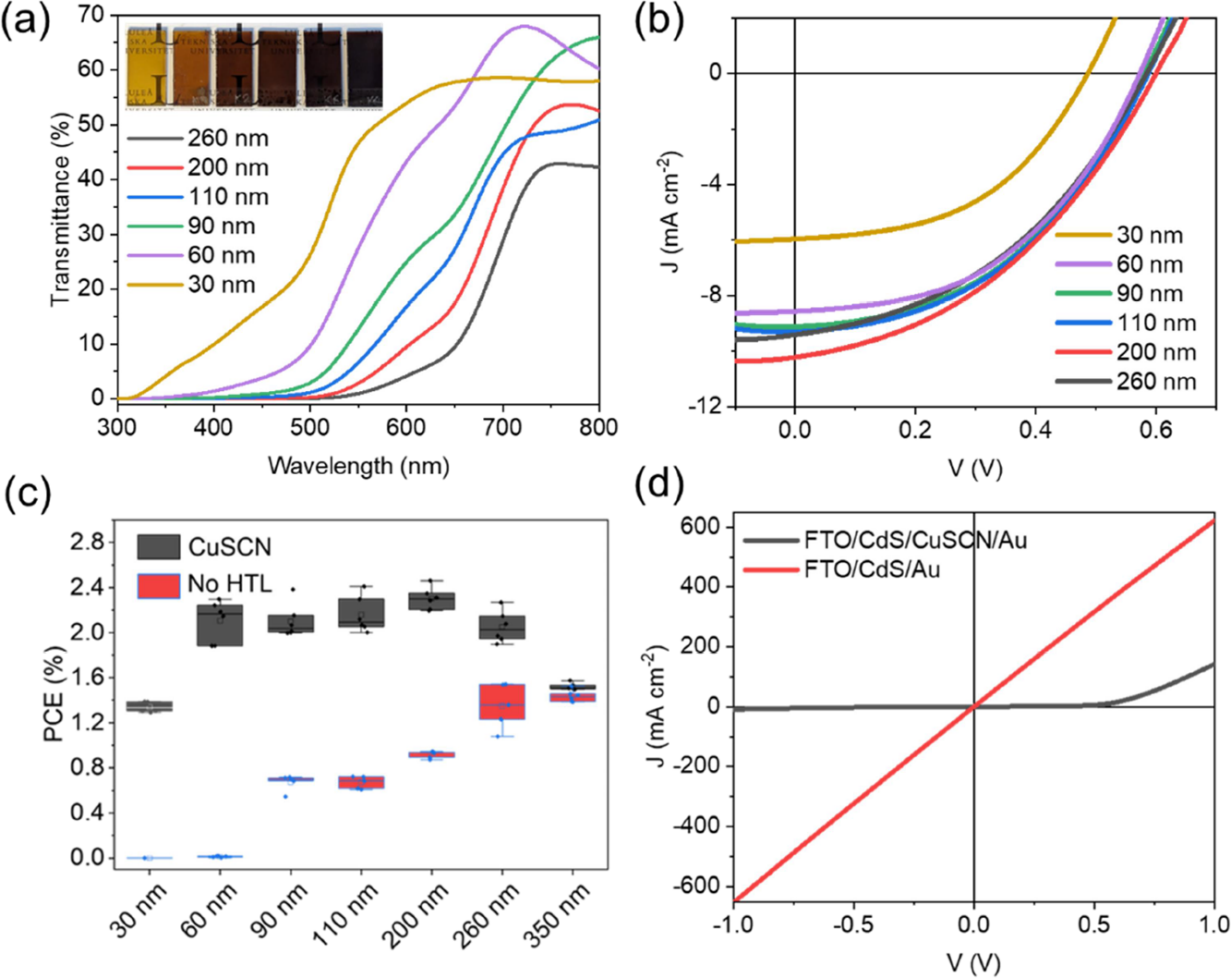
(a) Transmittance of FTO/CdS/Sb_2_S_3_/CuSCN stacks with varying thicknesses of the Sb_2_S_3_ absorber layer. The inset shows photographs of these stacks: the thickness of Sb_2_S_3_ increases from left (30 nm) to right (260 nm). (b) Performance parameters of devices (FTO/CdS/Sb_2_S_3_/CuSCN/Au) with varying thicknesses of absorber layers. (c) Thickness vs PCE variation of devices with and without CuSCN HTL layer. (d) *J–V* curves of devices representing direct contact between electron and hole contacts. FTO/CdS/Au and FTO/CdS/CuSCN/Au represent the pinhole areas (with no Sb_2_S_3_ layer) of the solar cells without and with the CuSCN HTL layer.

**Figure 6:**
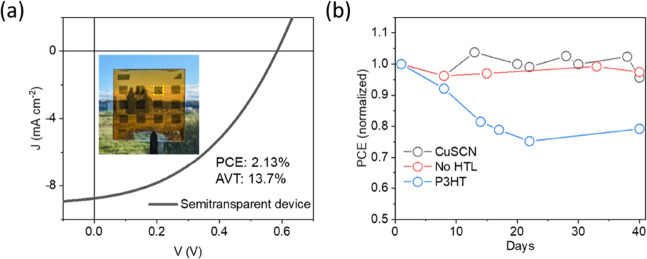
(a) *J–V* curve of a semitransparent Sb_2_S_3_ solar cell. The inset shows a photograph of the devices on a 25 × 25 mm^2^ substrate. The yellow dotted lines mark the device area (darker shade) with ∼10 nm Au electrode. (b) Variations of PCE in shelf-life stability testing of unencapsulated devices stored in the dark at an RH of 30–50% and a temperature of 20–26 °C.

Sb_2_S_3_ has a wide bandgap and high absorption coefficient (10^5^ cm^–1^); and therefore, it is a potential candidate for semitransparent and tandem solar cell applications.^32^ To this end, CuSCN with its high optical transparency (ultrawide bandgap of ∼3.9 eV) is favorably suitable as the HTL. To achieve sufficient semitransparency (of at least 25% for commercial applications), the thickness of the absorber layer must be reduced to less than 100 nm.^53^ The thickness of the absorber layer can be tuned by tuning the duration of the hydrothermal film deposition, as shown in Figure S11. The transmittance curves of the devices without the top contact layer (FTO/CdS/Sb_2_S_3_/CuSCN) are shown in Figure 5a, achieving high AVTs of 45.7 and 30.4% with 30 and 60 nm Sb_2_S_3_ absorber layers, respectively (AVT values are listed in Table S5). The *J*–*V* curves of the corresponding CuSCN-based devices with varying absorber thicknesses are shown in Figure 5b, and the performance parameters are listed in Table S5. The thickness reduction results in a higher probability of direct contact between electron and hole contacts via pinholes as shown in Figure S12 (arrows indicate the shunt paths in the ∼60 nm thick absorber layer). These shunt pathways lead to drastically decreased FF and *V*_OC_ in devices without CuSCN (as shown in Figure S13 and Table S6). P3HT has a similar shunt-reducing effect as seen in the PCE vs thickness trend in Figure S14a. The corresponding device performance parameters are listed in Table S7. The *J*–*V* curves (with varying Sb_2_S_3_ thicknesses) and cross-section SEM image of a typical P3HT device are shown in Figure S14b,c, respectively. As evident from the statistical distribution of PCEs vs thicknesses (Figure 5c), devices without the HTL layer did not perform at all until Sb_2_S_3_ thickness was increased to at least 90 nm. On the other hand, devices utilizing the CuSCN layer achieved a decent relative performance (compared to the device with the optimum thickness of 200 nm with a PCE of 2.46% as discussed earlier) with a PCE of 2.30% at 60 nm. Unfortunately, this pinhole shunt effect is not taken into account in the 1-D device simulation discussed earlier. The reason for the increased tolerance to shunts in ultrathin absorber layers can be found in the nature and magnitude of leakage currents in the regions where pinholes occur. Direct contact of CdS ETL with Au (FTO/CdS/Au) results in a very low shunt resistance (calculated from the slope at *V* = 0 V) of 1.6 Ω cm^2^ (Figure 5d). In contrast, it is increased by 2 orders of magnitude to 336 Ω cm^2^ upon insertion of a CuSCN layer (FTO/CdS/CuSCN/Au). Therefore, the parallel shunt current (created due to direct contact between the electron and hole contacts) is significantly more detrimental in the absence of the CuSCN layer. In addition, a nonohmic diode-like *J–V* characteristic for ETL-HTL devices has been reported to be more tolerant to shunts (pinholes) compared to an ohmic shunt path since the leakage currents are very low below the turn-on voltage (around 0.5 V).^68^ In summary, the CuSCN layer screens the detrimental effects of the two-dimensional variations in the thickness (and shunts) of the ultrathin Sb_2_S_3_ absorber film. A semitransparent proof-of-concept device with ultrathin Au electrode (∼10 nm based on the previous report)^53^ showed a PCE of 2.13% at an AVT of 13.7% (Figure 6a and Table S8). The cross-section SEM image is shown in Figure S15a, and the transmittance curves of the device with and without an Au electrode are shown in Figure S15b. The AVT of the device was reduced by more than 50% (from 30.4% for the 60 nm Sb_2_S_3_ absorber layer without the Au electrode) to 13.7% due to the low transmittance of the Au electrode.^53^ In comparison, due to the low AVT of P3HT (59.4%), FTO/CdS/Sb_2_S_3_ (60 nm)/P3HT shows an AVT of merely 9.0% (Figure S15b). This AVT loss can be reduced by employing an alternative top electrode with higher transparency (e.g., graphene as the transparent top electrode with more than 80% transmittance in the visible region, as reported by Zhang et al.^32^). The AVT of the device stack can be further improved by replacing CdS (AVT = 74.9%) with wide bandgap ETLs, such as TiO_2_ (AVT = 90.0%) (transmission spectra of these films are shown in Figure S3b).

### Stability

Solar cell devices integrating the CuSCN-based HTL show much better stability than those based on organic HTLs.^44,51^ The evolution performance parameters of devices without and with HTLs are shown in Figure 6b. The CuSCN-based device retained 96% of its initial PCE, after 40 days of ambient storage (similar to that without the HTL layer). In contrast, the P3HT-based device retained only 79% of its PCE. CuSCN is intrinsically stable and highly stable perovskite solar cells based on CuSCN have been reported frequently.^44,45^ CuSCN-based planar Sb_2_Se_3_ also showed excellent ambient shelf stability.^69^ However, despite these promising results, there is a need to investigate the photostability under continuous illumination under ambient (and controlled humid) conditions for these planar Sb_2_S_3_ solar cells.

## Conclusions

In summary, a low-temperature solution-processed HTL, CuSCN was utilized in hydrothermally deposited planar Sb_2_S_3_ solar cells. The optoelectronic properties of CuSCN for HTL applications were studied in detail. Material parameters influencing the hole transport characteristics of the CuSCN HTL and their effects on the device performance were simulated using drift-diffusion simulation software (SCAPS-1D). The simulation predicted increased *V*_OC_ and FF, with the addition of CuSCN as the HTL layer. Consistent with the simulation, in the devices, the CuSCN layer provided a high work function hole conducting-electron blocking layer between the Sb_2_S_3_ absorber and Au electrode and thus suppressed reverse leakage currents and improved recombination resistance. Devices with CuSCN HTL show a PCE ∼60% higher than those without the HTL (PCE of 2.46 vs 1.54%). CuSCN-based planar Sb_2_S_3_ devices have traditionally shown low PCEs in the limited number of reports in the literature, and this is one of the best-performing devices in the literature (Table S9) along with room for improvement via optimization of ETL and Sb_2_S_3_ layers. In addition, devices with semitransparent absorbers with thicknesses as low as 60 nm (AVT of 30.4% without top Au electrode) maintained decent performances of 2.30% in an opaque device and 2.13% in the corresponding semitransparent device (with AVT of 13.7% using ∼10 nm Au) because of the shunt-blocking properties of CuSCN HTL. These results show that CuSCN is a promising low-cost HTL candidate for all-inorganic planar semitransparent Sb_2_S_3_ solar cells based on a green and facile hydrothermal deposition.

## Supporting Information Available

SEM cross sections showing CuSCN thickness variations with spin coating speeds; details about SCAPS-1D simulation software; electrical parameters of layers used in SCAPS-1D simulation; literature reports of simulated and experimental results of planar Sb_2_S_3_ solar cells; a brief description of nonidealities and corresponding simulated solar cell performance parameters in ideal and nonideal cases for CuSCN, P3HT, and no-HTL devices; absorption/transmittance spectrum of Sb_2_S_3_, CuSCN, CdS, TiO_2_, and P3HT films; KPFM images and work function data of CuSCN and NiO films on FTO; cross-section image of hole-only SCLC device; SEM and AFM images of the top surfaces of Sb_2_S_3_ and Sb_2_S_3_/CuSCN; PCE performance variation vs thicknesses of CuSCN HTL; statistics of device performance parameters for devices with CuSCN, no-HTL, and P3HT; forward and reverse *J–V* curves of a typical CuSCN-based device; XPS and EDS spectra and analysis of Sb_2_S_3_ films; *J**–V* curves showing dark and light crossover; cross-section images of FTO/CdS/Sb_2_S_3_ stacks with varying hydrothermal deposition times (varying Sb_2_S_3_ thicknesses); cross-section images of CuSCN-based and no-HTL devices utilizing 60 nm Sb_2_S_3_ layers; *J**–V* curves of devices without HTL layers with varying Sb_2_S_3_ thicknesses; statistical distribution of PCE (with varying thicknesses of Sb_2_S_3_), *J**–V* curves (with varying thicknesses of Sb_2_S_3_) and a typical SEM cross-section image of P3HT-based devices; PCE variation with varying thicknesses of Sb_2_S_3_ absorber for CuSCN, P3HT, and no-HTL-based devices; typical SEM cross-section image of CuSCN-based semitransparent device; transmittance and AVTs of the semitransparent device along with FTO/CdS/Sb_2_S_3_/CuSCN or P3HT stacks without the top electrode; device performance parameters of semitransparent device; and literature reports of CuSCN-based planar solar cells (PDF)
