# Supplementary material for: All-Inorganic Hydrothermally Processed Semitransparent Sb2S3 Solar Cells with CuSCN as the Hole Transport Layer
Source: ACS Appl Energy Mater. 2024 Feb 5;7(4):1421–32. doi: 10.1021/acsaem.3c02492 (PMC10900181; doi:10.1021/acsaem.3c02492)
Supplement: Supplementary file 1 — ae3c02492_si_001.pdf [file ae3c02492_si_001.pdf]

Supplementary information for

All inorganic hydrothermally processed semitransparent Sb_2S_3 solar cells with CuSCN as the hole transport layer

Pankaj Kumar,[1*] Martin Eriksson,[1] Dzmitry S. Kharytonau[2], Shujie You,[1] Marta Maria Natile,[3,4] Alberto Vomiero[1,5*]

[1]Division of Materials Science, Department of Engineering Sciences and Mathematics, Luleå University of Technology, SE-971 87 Luleå, Sweden

[2]Electrochemistry and Corrosion Laboratory, Jerzy Haber Institute of Catalysis and Surface Chemistry, Polish Academy of Sciences, 30-239 Krakow, Poland

[3]Institute of Condensed Matter Chemistry and Technologies for Energy (ICMATE), National Research Council (CNR), via F. Marzolo 1, 35131 Padova, Italy.

[4]Department of Chemical Sciences, University of Padova, via F. Marzolo 1, 35131 Padova, Italy.

[5]Department of Molecular Sciences and Nanosystems, Ca' Foscari University of Venice, Via Torino 155, 30172 Venezia Mestre, Italy

*Corresponding authors

Email: pankaj.kumar@ltu.se , alberto.vomiero@ltu.se

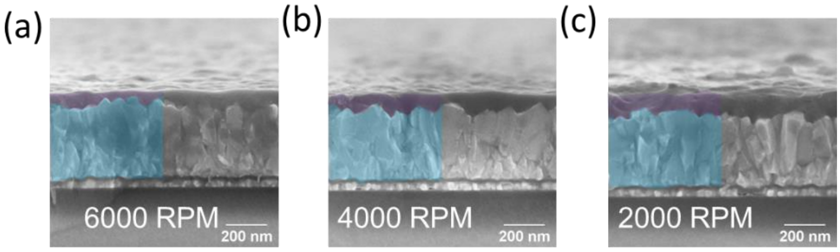

Figure S1. Variation of CuSCN thickness with spin coating speeds (RPMs).

Supplementary Note 1: SCAPS-1D Simulation Software

SCAPS-1D is a one-dimensional solar cell simulator developed by the Department of Electronics and Information Systems of the Gent University, Belgium. Its analysis is based on three basic semiconductor equations coupled with transport equations in one dimension (x, along the thickness) and in steady state conditions.[1]

Poisson's equation

$$\frac{\partial}{\partial x}\left(\varepsilon_0 \varepsilon \frac{\partial \Psi}{\partial x}\right) = -q\left(p - n + N_D - N_A + \rho_{def}\right) \quad (1)$$

Continuity equations

$$\frac{1}{q}\frac{\partial J_n}{\partial x} = U - G \tag{2}$$

$$\frac{1}{q}\frac{\partial J_p}{\partial x} = -(U - G) \tag{3}$$

where Ψ, q, ε, p/n, $N_{A/D}$ and ρ_{def} denote electrostatic potential, charge of an electron, dielectric permeability, hole/electron concentration, acceptor/donor density, and charge density defects, respectively. U is the recombination rate and G is the generation rate.

Drift induced transport equations

$$J_n = \frac{\mu_n n}{q}\frac{\partial E_{Fn}}{\partial x} \tag{4}$$

$$J_p = \frac{\mu_p p}{q}\frac{\partial E_{Fp}}{\partial x} \tag{5}$$

where $J_{p/n}$ represent current density of holes/electrons, $\mu_{p/n}$ represent hole/electron mobility, and $E_{Fp/Fn}$ specify the acceptor/donor fermi levels.

Table S1. Electrical parameters of FTO, CdS, Sb_2S_3, and CuSCN, used in SCAPS-1D simulation.

Parameters	FTO[2,3]	CdS[4]	Sb_2S_3[5]	CuSCN	P3HT[5]	CdS/Sb_2S_3 interface[b5]
CBM (eV)	4.4	4.0[6]	3.7	1.6[7]	3.2	
Bandgap (eV)	3.5	2.4	1.7	3.6	2.0	
Dielectric constant	9	10	7.1[8]	5.1[9]	3.0	
CB N_{eff} (cm^{-3})	2.2×10^{18}	2.2×10^{18}	5×10^{19}	2.2×10^{1810}	10^{20}	
VB N_{eff} (cm^{-3})	1.8×10^{19}	1.8×10^{19}	10^{20}	1.8×10^{1810}	10^{20}	
$\mu_{electron}$ (cm^2/V-s)	20	100	0.8[5]	1×10^{-4}	1×10^{-4}	
μ_{hole} (cm^2/V-s)	10	25	0.2[5]	1×10^{-4}	1×10^{-4}	
Doping, $N_{A/D}$ (cm^{-3})	10^{19}	1.1×10^{18}	7.3×10^{1311}	5.3×10^{17}	1×10^{17}	
Defect type[a]	-	Acceptor	Neutral		Neutral	Acceptor
Defect density (cm^{-3})	-	1×10^{18}	1×10^{12}	10^{1412}	10^{16}	10^{1213}
Defect capture cross-section (cm^{-2})	-	10^{-17} (n)<br>10^{-12} (p)	10^{-14} (n)<br>5×10^{-15} (p)	10^{-15} (p)<br>10^{-15} (p)	10^{-16} (p)	10^{-13} (n)
Defect energetic position (eV)	-	-	$E_v + 0.52$	$E_v + 0.90$[14]	$E_V + 0.35$	$E_c - 1.0$
Thickness (nm)	50	70	350	50	50	NA
Series Resistance: 1 Ω cm^2<br>Shunt Resistance: 1000 Ω cm^2						
Electrodes	Left contact [FTO]			Right contact [Au]		
Work function/alignment	Flat band			5.1 eV		
SRV, electrons	10^7			10^5		
SRV, holes	10^5			10^5		
Tunneling	None			None		
Reflection	None			None		

[a]Defect-related parameters for Sb_2S_3 were taken from Kondrotas et al.[5] and Myagmarsereejid et al.[15]; [b]CdS/Sb_2S_3 interface defect was only used in non-ideal case (Case C) discussed later in Supplementary Note 2.

CBM: Conduction band maximum; CB/VB N_{eff}: conduction band/valence band effective density of states; μ: mobility; SRV: Surface recombination velocity.

Table S2. Comparison table for reported simulations and experimental performances of Sb_2S_3 based planar devices.

Device	V_{OC} [V]	J_{SC} [mA cm^{-2}]	FF [%]	PCE [%]	Ref
FTO/TiO_2/Sb_2S_3/P3HT/PEDOT:PSS/Au	1.3	19.0	81	20.00	[5]
FTO/TiO_2/Sb_2S_3/V_2O_5/Au	1.16	21.12	80.7	19.74	[16]
FTO/WS_2/Sb_2S_3/Au	1.23	24.0	89.6	26.6	[1]
Au/Sb_2S_3/ZnS/front contact	1.09	24.6	80.6	21.7	[8]
Au/Sb_2S_3/CdS/ZnO/ZnO:Al	0.72	15.98	83.23	9.51	[17]
Au/Sb_2S_3/CdS/ZnO/AZO	1.23	20.14	83.1	20.6	[18]
FTO/ZnS/Sb_2S_3/Cu_2O/Au	0.97	23.73	72.32	16.65	[19]
SQ limit	1.398	23.06	90.87	29.28	[20]
Experimental reports in literature					
FTO/CdS/Sb_2S_3/ Spiro-OMeTAD /Au	0.76	17.41	60.48	8.00	[21]
FTO/SnO_2/CdS/Sb_2S_3/ Spiro-OMeTAD /Au	0.796	17.57	58.16	7.21	[22]
FTO/CdS/Sb_2S_3/P3HT/Au	0.773	14.67	54.79	6.21	[23]
FTO/CdS/Sb_2S_3/Spiro-OMeTAD/Au	0.805	12.79	63.71	6.56	[24]

Supplementary Note 2: Applying non-ideal conditions to devices simulated using parameters from **Table S1**.

The efficiencies of devices simulated in SCAPS-1D using parameters in **Table S1** ('ideal' conditions) (including most of the optimized simulated devices in literature listed in **Table S2**) were quite high as compared to the record PCE of 8% for Sb_2S_3 based solar cell.[21] Therefore, a series of realistic conditions were applied to the 'ideal' case devices based on experimental data and literature.

Case A: Update the actual thickness of absorber layers and add obtained R_s and R_{sh} values from champion devices:

The optimum thickness of Sb_2S_3 absorber champion devices with CuSCN (200 nm), P3HT (150 nm) and without HTL (260 nm) layers were updated from the ideal case thickness of 350 nm (based on the average of Sb_2S_3 layer thicknesses from reported devices based on hydrothermal deposition).[23–28] The series and shunt resistances were also added. For the ideal cases, R_s = 1 Ω cm^2 and R_{sh} = 1000 Ω cm^2 were used (**Table S1**). These values were updated with the actual values in **Table 1** of the main article.

Since the optimum thicknesses of absorber layers in the three devices were lower, J_{SC} values were reduced from around 16 mA cm^{-2} to 11-12 mA cm^{-2} in all cased. FF decreased considerably with the introduction of actual resistances while V_{OC}'s remained almost the same in all three devices. (Table S2 and Figure S1).

Case B: Add deep defects in the absorber layer:

The presence of deep defects in Sb_2S_3 thin films has been widely reported.[5,21,29] Oxide impurities and sulfur vacancy as seen in XPS and EDS data (shown below in **Figure S8** and **Figure S9**) form deep traps too.[30] Therefore, the defect concentration was updated to 5 × 10^{14} cm^{-3} as reported by Choi et. al.[31] These deep defects directly affect the V_{OC} of Sb_2S_3 solar cells and can be seen in the simulations too.[13]

Case C: Add interface defects at CdS/Sb_2S_3 interface:

Defects at the CdS/Sb_2S_3 interface can also limit the V_{OC}.[13,32,33] Interface defect at CdS/Sb_2S_3 interface was set to 10^{12} cm^{-2} in the same order as reported by Zeng et al.[13]

Comments on the experimental vs simulated J-V data: As shown in **Table S3** and **Figure S2**, there remain significant deviations between simulated and experimental data, especially in the V_{OC}'s. The simulations can be further improved by applying experimental data to introduce surface defects at the Sb_2S_3/HTL interfaces and at the Au contacts (Fermi level pinning due to metal induced defects) and band tails in the Sb_2S_3 layer.[5,34] P3HT has been reported to form passivating contact (bonding interaction) with Sb_2S_3 while there is no such report for CuSCN/Sb_2S_3 interface.[23,35] Also, simulations parameters are mostly taken from reference and therefore the device performance parameters should be useful mainly for comparative studies.

It is important to highlight that the SCAPS-1D simulations has several limitations such as (a) lack of accounting for grain boundary and surface discontinuities on surfaces and (b) inability to include reflection and interference at all interfaces. The light intensity distribution is expected to be different in the three devices because of the optical spacer effects due to CuSCN and P3HT.

Table S3: Comparison table of simulations for three non-ideal cases discussed above (in Supplementary note 2).

HTL	Case	J_{SC} [mA cm^{-2}]	V_{OC} [V]	FF [%]	PCE [%]
CuSCN	Ideal	16.04	1.34	76.19	16.48
	Case A	11.62	1.35	53.57	8.40
	Case B	11.52	1.05	48.58	5.91
	Case C	11.53	0.82	43.58	4.12
	Experimental	10.22	0.60	40.13	2.46
No HTL	Ideal	15.91	1.05	70.08	11.75
	Case A	12.06	0.99	39.01	4.7
	Case B	11.9	0.97	37.40	4.34
	Case C	11.76	0.76	35.42	3.16
	Experimental	8.36	0.45	40.36	1.54
P3HT	Ideal	16.22	1.35	76.91	16.84
	Case A	10.69	1.35	52.91	7.66
	Case B	10.62	1.05	50.25	5.59
	Case C	10.65	0.81	47.89	4.15
	Experimental	10.34	0.68	48.41	3.42

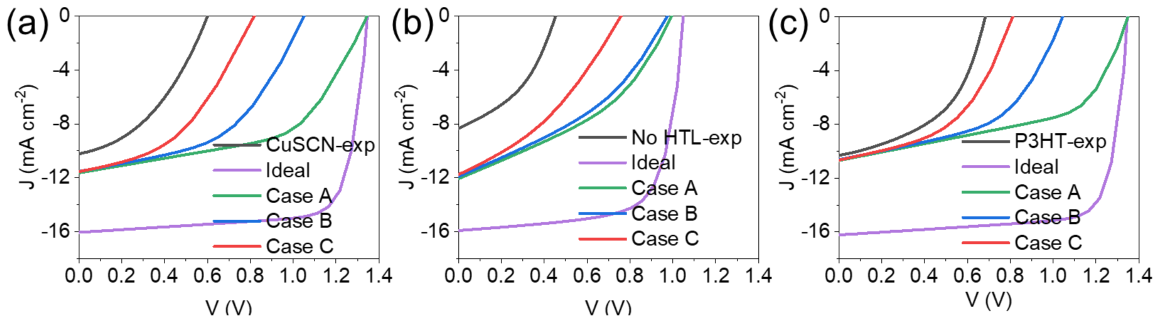

Figure S2. J-V curves for non-ideal conditions for Case A, Case B, and Case C, as per **supplementary note 2** using (a) CuSCN as HTL, (b) Without HTL, and (c) P3HT as HTL. The corresponding experimental J-V data is also included for comparison.

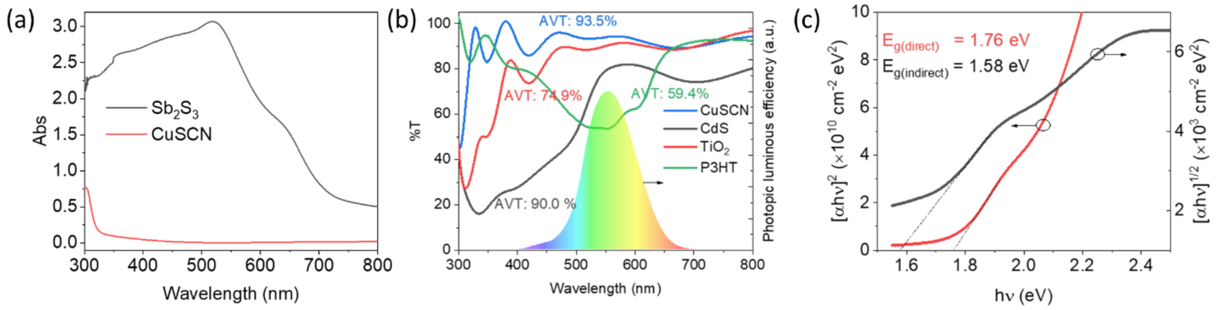

Figure S3. (a) Absorption spectrum of Sb_2S_3 and CuSCN films used in the solar cell devices. (b) Transmittance and AVTs of thin films on FTO glass. The photopic luminous efficiency which represents the relative sensitivity of the human eye vs. wavelength is also shown. (c) Tauc plots of Sb_2S_3 films used in direct and indirect optical bandgap calculation.

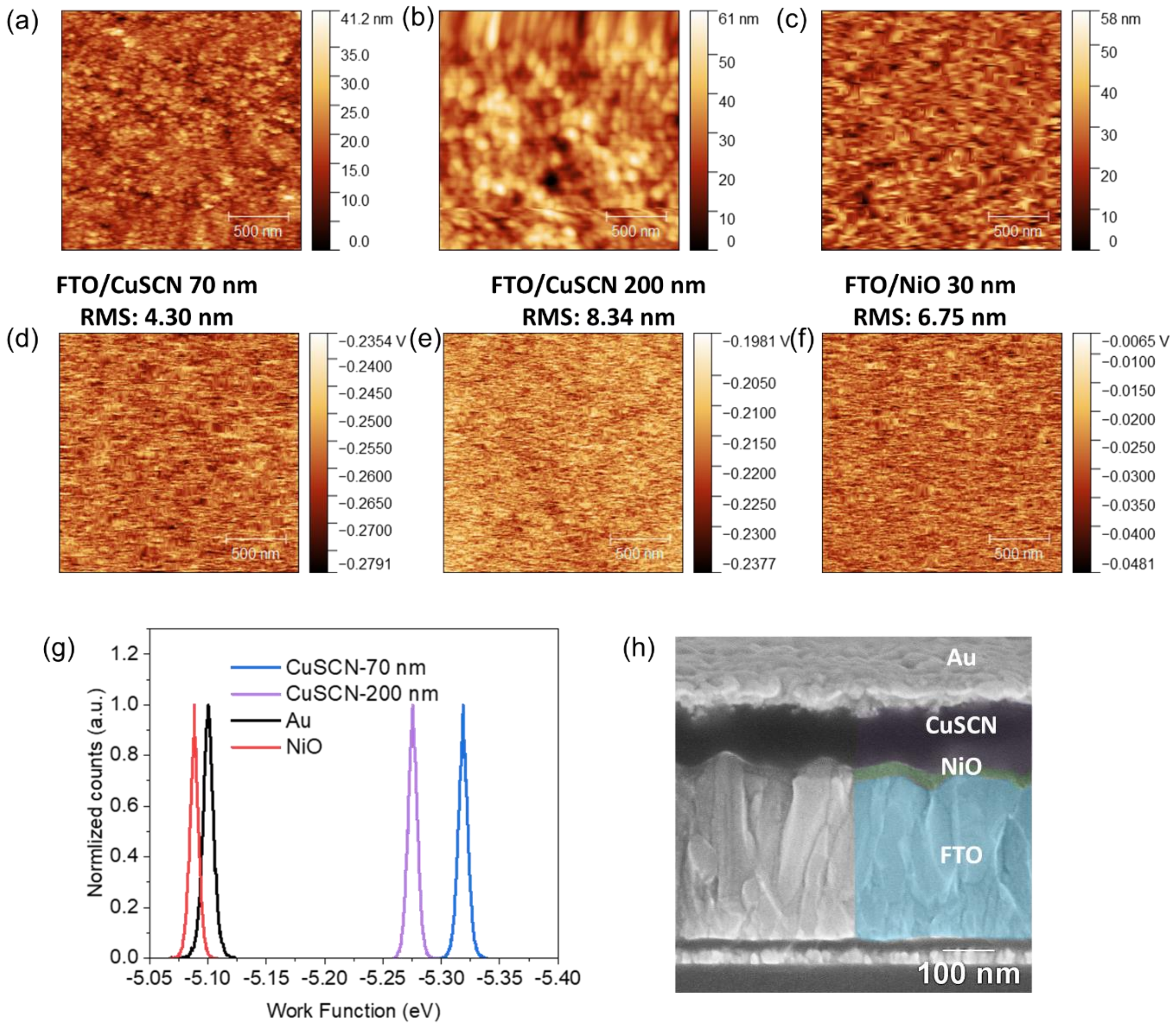

Figure S4. AFM morphology of (a) 70 nm CuSCN (b) 200 nm CuSCN (c) NiO thin films on FTO. Simultaneously acquired contact potential difference (CPD) maps (d)-(f), corresponding to (a)-(c). Work function (WF) histogram plots of NiO, CuSCN (70 nm), and CuSCN (200 nm) films on FTO. Au on FTO glass was used for calibration with mean WF of -5.1 eV. (h) Cross Sectional SEM image of typical SCLC device using NiO (20-30 nm) as hole selective contact.

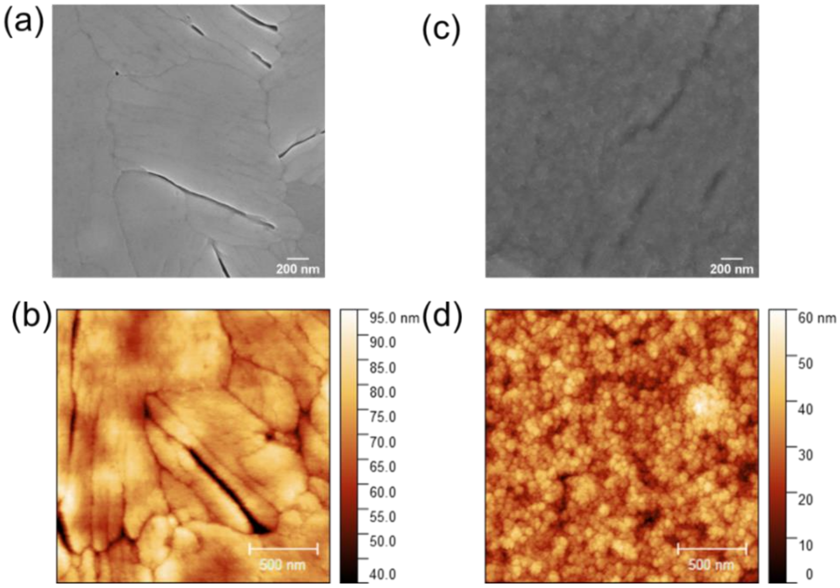

Figure S5. Top surface SEM of (a) Sb_2S_3, (c) Sb_2S_3/CuSCN; corresponding AFM images are shown in (b) and (d), respectively.

Table S4. Device performance parameters of solar cell devices with varying thicknesses of the CuSCN layer for Sb_2S_3 hydrothermal deposition time fixed at 1 hr 45 min.

CuSCN RPMs	CuSCN thickness [nm][a]	V_{OC} [V]	J_{SC} [mA cm^{-2}]	FF [%]	PCE [%]
2000	100	0.58 ± 0.01 (0.56)	8.67 ± 0.39 (9.25)	38.72 ± 1.32 (40.77)	1.94 ± 0.10 (2.12)
4000	60	0.59 ± 0.01 (0.59)	8.57 ± 0.58 (9.29)	42.99 ± 1.04 (44.20)	2.16 ± 0.15 (2.41)
6000	35	0.53 ± 0.01 (0.53)	7.19 ± 0.65 (7.96)	42.31 ± 0.99 (42.31)	1.60 ± 0.11 (1.79)

[a]Measured average thickness on FTO substrates, as shown in Figure S1.

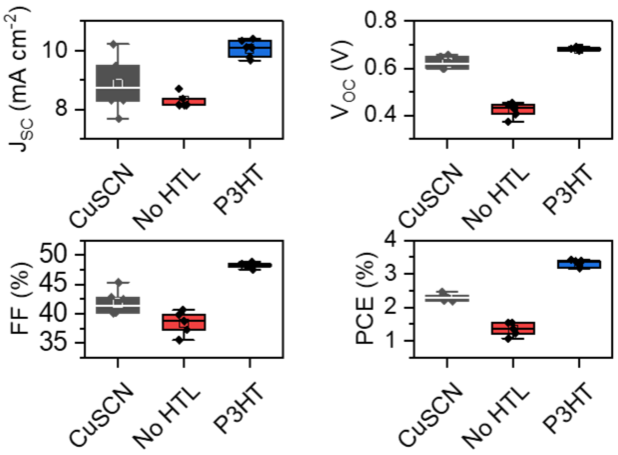

Figure S6. Statistical distribution of device parameters for Sb_2S_3 devices without HTL and P3HT and CuSCN HTLs. The absorber and HTL thicknesses of these optimum devices for each group are given in Table 1 footnotes (main article).

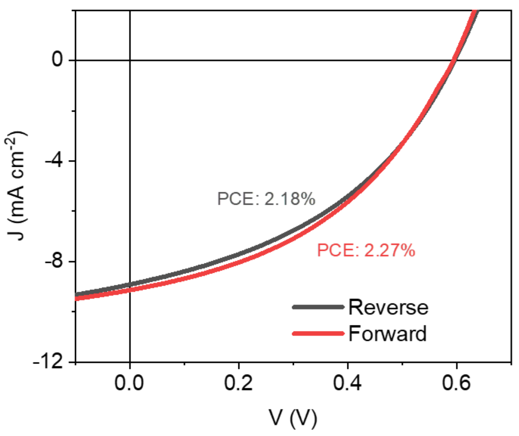

Figure S7. Forward and reverse scan of a typical solar cell based on CuSCN HTL. Forward scan: -0.2 V -> 0.8 V, reverse scan: 0.8 V -> -0.2 V. Scan speed: ~100 mV/s.

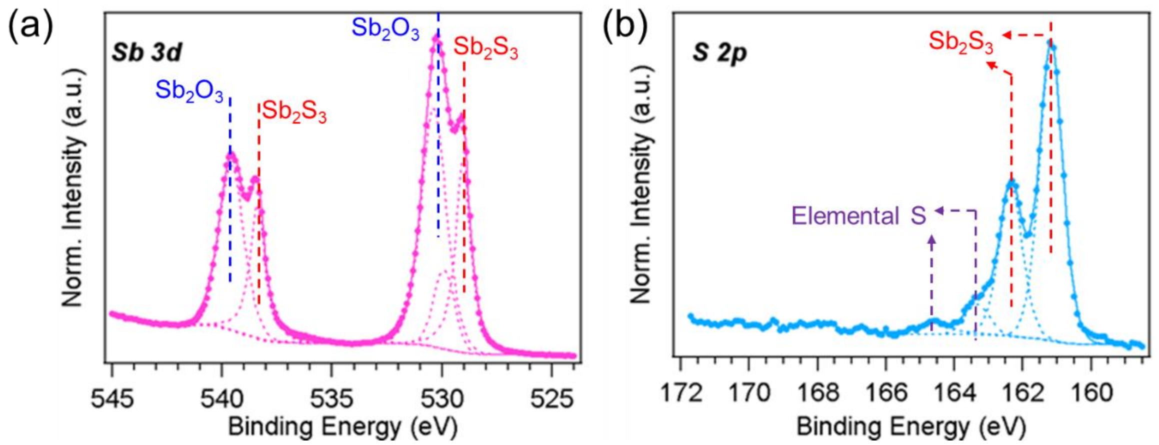

Figure S8. XPS spectra of (a) Sb 3d and (b) S 2p regions of hydrothermally deposited and annealed Sb_2S_3 film.

Supplementary Note 3. XPS analysis for **Figure S8**:

The analysis of Sb 3d core level reveals the presence on the surface of two different Sb species. The doublet Sb $3d_{5/2}$ and $3d_{3/2}$ at 529.3 and 538.7 eV is characteristic of Sb^{3+} in Sb_2S_3, while the one at higher BEs (530.7 and 539.9 eV) agrees with Sb^{3+} in Sb_2O_3.[31,36,37] This last presence is confirmed by a contribution at 530.2 eV characteristic of O1s XPS peak.[38] The relative atomic % of Sb^{3+}-O results higher than that of Sb^{3+}-S (63.2 % vs 36.8 %). The presence of Sb_2O_3 could be due to the residual impurities in the tubular chamber used for annealing or subsequent exposure to air.[39] The fitting of S 2p core level shows two characteristic doublets: the most intense at lower BEs (161.2 and 162.3 eV for S $2p_{3/2}$ and $2p_{1/2}$, respectively) is ascribable to sulphur in Sb_2S_3.[36,37] The small doublet at higher BEs (163.4 and 164.6 eV for S $2p_{3/2}$ and $2p_{1/2}$, respectively) suggests the presence of elemental sulphur.[36] The sulfur (a polyatomic nonmetal) at the surface can act as resistive barrier for charges.[31]

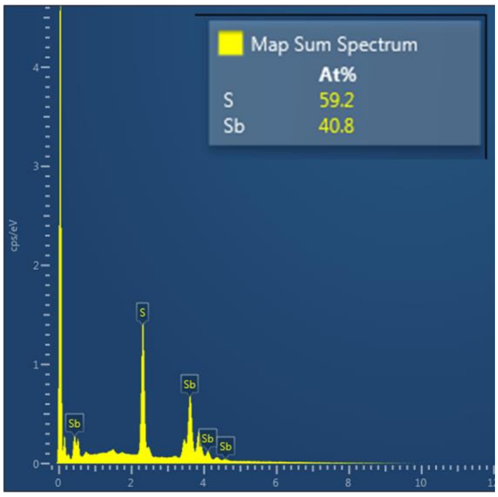

Figure S9. EDS spectrum of the annealed Sb_2S_3 thin films hydrothermally deposited on FTO.

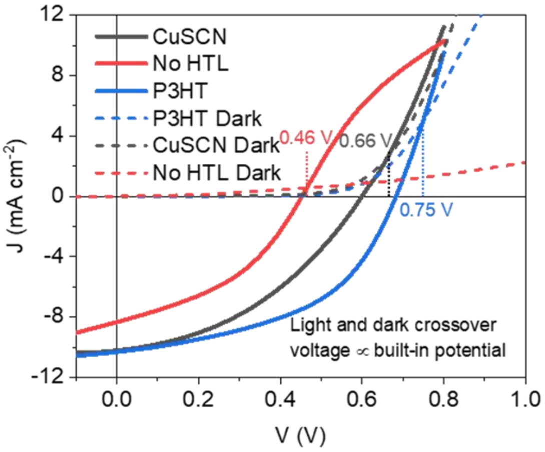

Figure S10. J-V curves showing light-dark crossover voltages which are proportional to the built-in voltage in the respective devices.

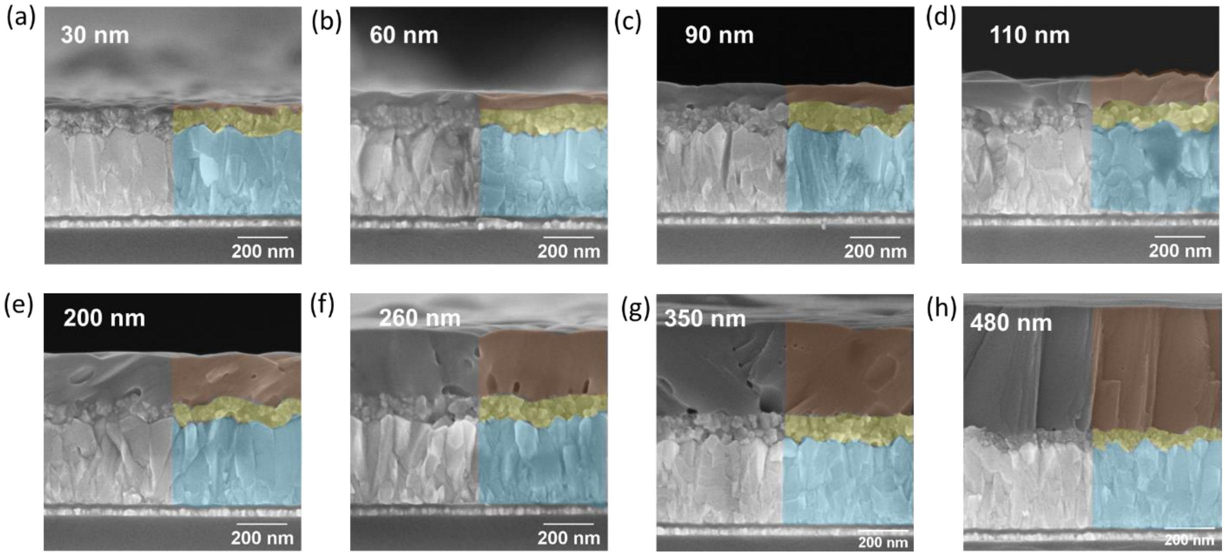

Figure S11. The cross-section SEM images of Sb_2S_3 films with hydrothermal deposition times of (a) 60 min, (b) 75 min, (c) 90 min (d) 105 min, (e) 120 min, (f) 150 min, (g) 180 min, and (h) 240 min, respectively.

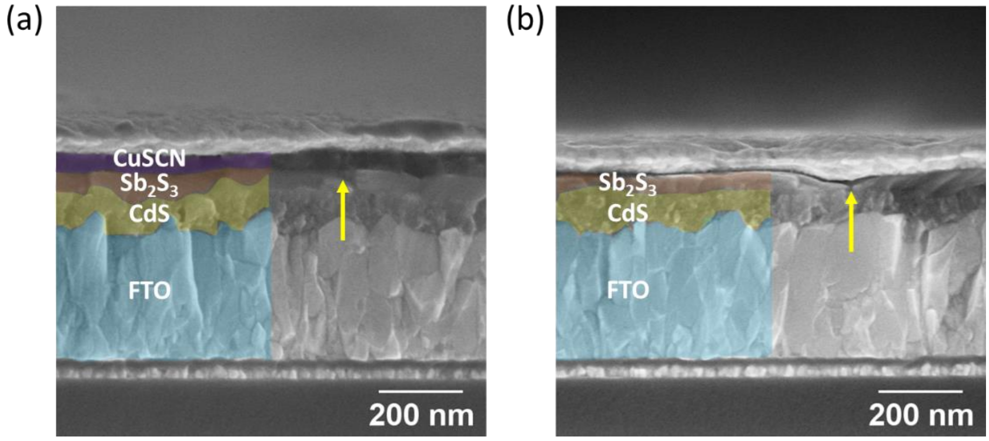

Figure S12. SEM cross sections of devices utilizing ultrathin (~60 nm) Sb_2S_3 layers (a) with CuSCN layer and (b) without CuSCN HTL. The yellow arrows point to the possible shunt points.

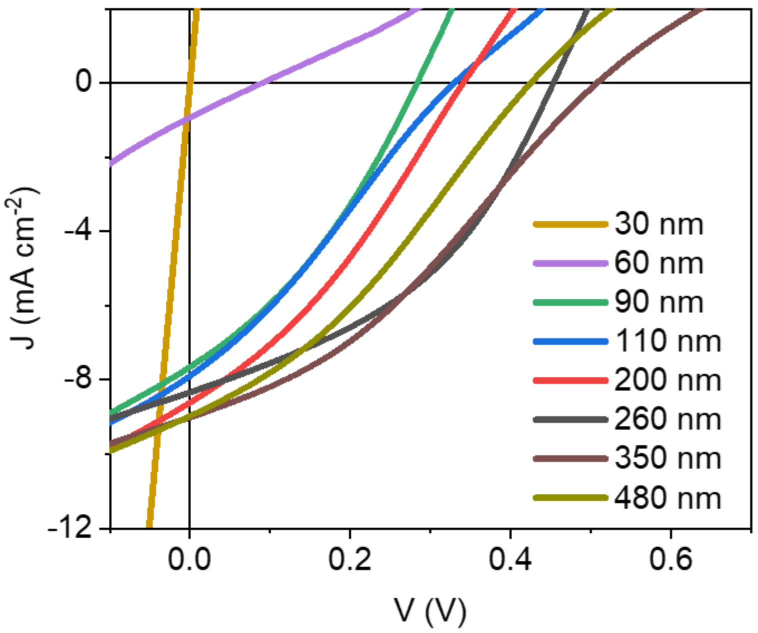

Figure S13. J-V curves of devices with varying thicknesses of the Sb_2S_3 layer without the HTL layer.

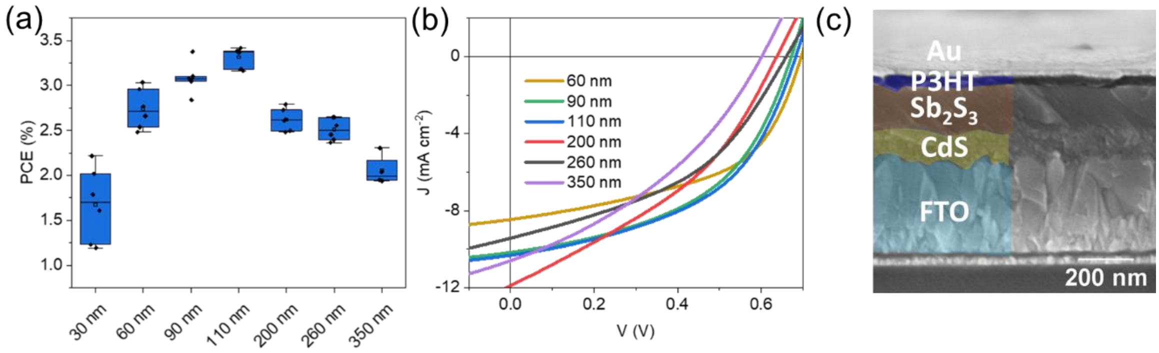

Figure S14. (a) Statistical distribution of PCE with varying thicknesses of the Sb_2S_3 layer with the P3HT HTL layer. (b) J-V curves of devices with varying thicknesses of the Sb_2S_3 layer with the P3HT HTL layer. (c) Cross-section SEM image of a typical P3HT based device.

Table S5 Device performance parameters of solar cell devices with CuSCN HTL, with varying thicknesses of Sb_2S_3 absorber layer.

HT time [min]	V_{OC} [V]	J_{SC} [mA cm^{-2}]	FF [%]	PCE [%]	Sb_2S_3 thickness [nm]	AVT without Au electrode*
180	0.64 ± 0.01 (0.63)	5.92 ± 0.15 (5.86)	40.46 ± 1.84 (42.24)	1.52 ± 0.03 (1.57)	350	-
150	0.58 ± 0.01 (0.58)	8.85 ± 0.63 (9.42)	39.69 ± 1.82 (41.23)	2.05 ± 0.13 (2.27)	260	2.3

120	0.62 ± 0.02 (0.60)	8.86 ± 0.85 (10.22)	41.86 ± 1.89 (40.13)	2.30 ± 0.09 (2.46)	200	5.2
105	0.59 ± 0.01 (0.59)	8.57 ± 0.58 (9.29)	42.99 ± 1.04 (44.20)	2.16 ± 0.15 (2.41)	110	9.9
90	0.57 ± 0.01 (0.58)	8.90 ± 0.59 (9.11)	41.53 ± 2.67 (45.06)	2.09 ± 0.14 (2.38)	90	15.8
75	0.58 ± 0.01 (0.57)	8.23 ± 0.43 (8.57)	44.25 ± 2.84 (46.81)	2.11 ± 0.14 (2.30)	60	30.4
60	0.49 ± 0.00 (0.49)	5.61 ± 0.20 (5.97)	49.00 ± 1.60 (47.86)	1.34 ± 0.04 (1.39)	30	45.7

*AVT of device layers without top electrode (FTO/CdS/Sb_2S_3/CuSCN). Parameters of champion devices are included in parenthesis.

Table S6. Device performance parameters of solar cell devices without HTL, with varying thicknesses of Sb_2S_3 absorber layer.

HT time [min]	V_{OC} [V]	J_{SC} [mA cm-2]	FF [%]	PCE [%]	Sb_2S_3 thickness [nm]
240	0.42 ± 0.08 (0.43)	8.29 ± 0.41 (9.05)	29.57 ± 1.06 (31.82)	1.03 ± 0.10 (1.23)	480
180	0.49 ± 0.03 (0.51)	9.07 ± 0.66 (9.04)	32.76 ± 0.46 (33.07)	1.45 ± 0.05 (1.53)	350
150	0.42 ± 0.03 (0.45)	8.30 ± 0.22 (8.36)	38.40 ± 1.84 (40.63)	1.35 ± 0.18 (1.54)	260
120	0.33 ± 0.01 (0.34)	8.58 ± 0.13 (8.66)	32.59 ± 0.41 (31.95)	0.92 ± 0.03 (0.94)	200
105	0.31 ± 0.02 (0.33)	7.70 ± 0.18 (7.95)	28.11 ± 1.25 (27.42)	0.67 ± 0.05 (0.72)	110
90	0.27 ± 0.02 (0.28)	7.75 ± 0.26 (7.70)	0.32 ± 0.79 (33.02)	0.67 ± 0.06 (0.72)	90
75	0.06 ± 0.02 (0.09)	0.89 ± 0.08 (0.97)	22.18 ± 1.04 (23.26)	0.01 ± 0.01 (0.02)	60
60	0.00	0.95	-	0.00	30

Table S7. Device performance parameters of solar cell devices with P3HT HTL, with varying thicknesses of Sb_2S_3 absorber layer.

HT time [min]	V_{OC} [V]	J_{SC} [mA cm^{-2}]	FF [%]	PCE [%]	Sb_2S_3 thickness [nm]
180	0.59 ± 0.02 (0.60)	9.90 ± 0.60 (10.65)	35.40 ± 0.66 (36.02)	2.06 ± 0.15 (2.30)	350

150	0.65 ± 0.00 (0.66)	9.15 ± 0.25 (9.47)	41.86 ± 0.70 (42.39)	2.51 ± 0.11 (2.65)	260
120	0.63 ± 0.00 (0.64)	11.41 ± 0.44 (11.97)	36.27 ± 0.50 (36.57)	2.62 ± 0.11 (2.79)	200
105	0.68 ± 0.00 (0.68)	10.07 ± 0.27 (10.34)	48.24 ± 0.40 (48.41)	3.31 ± 0.10 (3.42)	110
90	0.68 ± 0.00 (0.67)	9.74 ± 0.29 (10.21)	46.85 ± 1.69 (49.50)	3.08 ± 0.16 (3.37)	90
75	0.69 ± 0.08 (0.69)	8.15 ± 0.29 (8.48)	48.83 ± 2.53 (51.44)	2.74 ± 0.20 (3.03)	60
60	0.65 ± 0.02 (0.68)	5.61 ± 0.38 (6.28)	45.56 ± 6.68 (52.15)	1.68 ± 0.38 (2.22)	30

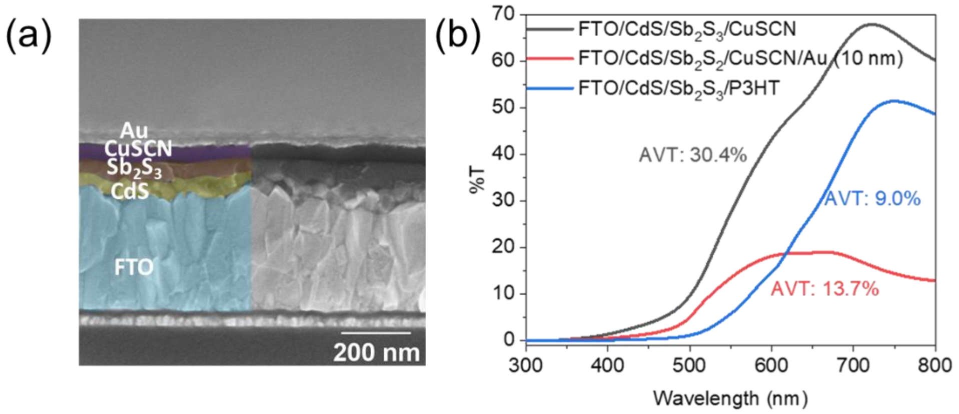

Figure S15. (a) Cross-section SEM image of semitransparent solar cell with ~10 nm Au electrode. (b) Transmittance and AVT of the semitransparent device (10 nm Au) using CuSCN HTL (60 nm Sb_2S_3) along with FTO/CdS/Sb_2S_3/CuSCN or P3HT stacks without the top electrode.

Table S8 Device performance parameters of semitransparent solar cell devices with 60 nm Sb_2S_3 absorber layer and ~10 nm ultrathin Au layer.

V_{OC} [V]	J_{SC} [mA cm^{-2}]	FF [%]	PCE [%]	Sb_2S_3 thickness [nm]	AVT of device [%]
0.59 ± 0.02 (0.58)	7.85 ± 0.69 (8.76)	43.60 ± 1.39 (41.72)	2.02 ± 0.08 (2.13)	60	13.7

Table S9. Literature reports of planar solution processed Sb_2S_3 solar cells with CuSCN as HTL.

Electrode	CuSCN deposition	Sb_2S_3 deposition	V_{OC} [V]	J_{SC} [mA cm^{-2}]	FF [%]	PCE [%]	Ref
Au	Spin coating	CBD	0.46	5.44	0.33	0.80	[40]
Au	Spin coating	Thermal evaporation	0.60	6.43	0.44	1.69	[40]
Au	Impregnation	CBD	0.48	4.85	0.38	0.90	[41]
Au	Impregnation	Sputtering	0.59	5.26	0.54	1.67	[41]
Au	Wiping	CBD	0.55	4.30	0.35	0.83	[42]
Carbon/Ag	Spin coating	CBD	0.61	6.25	0.51	1.95	[43]
Ag	Spin coating	Hydrothermal	0.29	6.01	0.31	0.55	[44]
Au	Spin coating	Hydrothermal	0.60	10.22	0.40	2.46	This work

CBD: Chemical bath deposition

References:

(1) Rahman, Md. F.; Alam Moon, Md. M.; Hossain, M. K.; Ali, Md. H.; Haque, Md. D.; Kuddus, A.; Hossain, J.; Md. Ismail, A. B. Concurrent Investigation of Antimony Chalcogenide (Sb2Se3 and Sb2S3)-Based Solar Cells with a Potential WS2 Electron Transport Layer. *Heliyon* **2022**, *8* (12), e12034. https://doi.org/10.1016/j.heliyon.2022.e12034.

(2) Ahmed, S.; Jannat, F.; Khan, Md. A. K.; Alim, M. A. Numerical Development of Eco-Friendly Cs2TiBr6 Based Perovskite Solar Cell with All-Inorganic Charge Transport Materials via SCAPS-1D. *Optik* **2021**, *225*, 165765. https://doi.org/10.1016/j.ijleo.2020.165765.

(3) Kuddus, A.; Rahman, M. F.; Ahmmed, S.; Hossain, J.; Ismail, A. B. M. Role of Facile Synthesized V2O5 as Hole Transport Layer for CdS/CdTe Heterojunction Solar Cell: Validation of Simulation Using Experimental Data. *Superlattices Microstruct.* **2019**, *132*, 106168. https://doi.org/10.1016/j.spmi.2019.106168.

(4) Nicolás-Marín, M. M.; Vigil-Galán, O.; Ayala-Mato, F.; Courel, M. Analysis of Hole Transport Layer and Electron Transport Layer Materials in the Efficiency Improvement of Sb2(Se1−x S x)3 Solar Cell. *Phys. Status Solidi B* **2023**, *260* (1), 2200342. https://doi.org/10.1002/pssb.202200342.

(5) Kondrotas, R.; Chen, C.; Tang, J. Sb2S3 Solar Cells. *Joule* **2018**, *2* (5), 857–878. https://doi.org/10.1016/j.joule.2018.04.003.

(6) Ngoupo, A. T.; Ouédraogo, S.; Zougmoré, F.; Ndjaka, J. M. B. Numerical Analysis of Ultrathin Sb2Se3-Based Solar Cells by SCAPS-1D Numerical Simulator Device. *Chin. J. Phys.* **2021**, *70*, 1–13. https://doi.org/10.1016/j.cjph.2020.12.010.

(7) Wijeyasinghe, N.; Eisner, F.; Tsetseris, L.; Lin, Y.-H.; Seitkhan, A.; Li, J.; Yan, F.; Solomeshch, O.; Tessler, N.; Patsalas, P.; Anthopoulos, T. D. P-Doping of Copper(I) Thiocyanate (CuSCN) Hole-Transport Layers for High-Performance Transistors and Organic Solar Cells. *Adv. Funct. Mater.* **2018**, *28* (31), 1802055. https://doi.org/10.1002/adfm.201802055.

(8) Islam, M. T.; Thakur, A. K. Two Stage Modelling of Solar Photovoltaic Cells Based on Sb2S3 Absorber with Three Distinct Buffer Combinations. *Sol. Energy* **2020**, *202*, 304–315. https://doi.org/10.1016/j.solener.2020.03.058.

(9) Pattanasattayavong, P.; Mottram, A. D.; Yan, F.; Anthopoulos, T. D. Study of the Hole Transport Processes in Solution-Processed Layers of the Wide Bandgap Semiconductor Copper(I) Thiocyanate (CuSCN). *Adv. Funct. Mater.* **2015**, *25* (43), 6802–6813. https://doi.org/10.1002/adfm.201502953.

(10) Madhavan, V. E.; Zimmermann, I.; Baloch, A. A. B.; Manekkathodi, A.; Belaidi, A.; Tabet, N.; Nazeeruddin, M. K. CuSCN as Hole Transport Material with 3D/2D Perovskite Solar Cells. *ACS Appl. Energy Mater.* **2020**, *3* (1), 114–121. https://doi.org/10.1021/acsaem.9b01692.

(11) Liu, M.; Gong, Y.; Li, Z.; Dou, M.; Wang, F. A Green and Facile Hydrothermal Approach for the Synthesis of High-Quality Semi-Conducting Sb2S3 Thin Films. *Appl. Surf. Sci.* **2016**, *387*, 790–795. https://doi.org/10.1016/j.apsusc.2016.06.126.
(12) Tara, A.; Bharti, V.; Sharma, S.; Gupta, R. Device Simulation of FASnI3 Based Perovskite Solar Cell with Zn(O0.3, S0.7) as Electron Transport Layer Using SCAPS-1D. *Opt. Mater.* **2021**, *119*, 111362. https://doi.org/10.1016/j.optmat.2021.111362.
(13) Zeng, Y.; Huang, J.; Li, J.; Sun, K.; Shah, U. A.; Deng, H.; Zhang, X.; Sha, C.; Qian, C.; Song, H.; Hao, X. Comparative Study of TiO2 and CdS as the Electron Transport Layer for Sb2S3 Solar Cells. *Sol. RRL* **2022**, *n/a* (n/a), 2200435. https://doi.org/10.1002/solr.202200435.
(14) Jaffe, J. E.; Kaspar, T. C.; Droubay, T. C.; Varga, T.; Bowden, M. E.; Exarhos, G. J. Electronic and Defect Structures of CuSCN. *J. Phys. Chem. C* **2010**, *114* (19), 9111–9117. https://doi.org/10.1021/jp101586q.
(15) Myagmarsereejid, P.; Ingram, M.; Batmunkh, M.; Zhong, Y. L. Doping Strategies in Sb2S3 Thin Films for Solar Cells. *Small* **2021**, *17* (39), 2100241. https://doi.org/10.1002/smll.202100241.
(16) Singh, V. K.; Srivastava, S.; Singh, A. K.; Chauhan, M. S.; Patel, S. P.; Singh, R. S. Theoretical Study of Highly Efficient All-Inorganic Sb2S3-on-Si Monolithically Integrated (2-T) and Mechanically Stacked (4-T) Tandem Solar Cells Using SCAPS-1D. *Environ. Sci. Pollut. Res.* **2023**. https://doi.org/10.1007/s11356-023-25292-2.
(17) Basak, A.; Singh, U. P. Numerical Modelling and Analysis of Earth Abundant Sb2S3 and Sb2Se3 Based Solar Cells Using SCAPS-1D. *Sol. Energy Mater. Sol. Cells* **2021**, *230*, 111184. https://doi.org/10.1016/j.solmat.2021.111184.
(18) Courel, M.; Jiménez, T.; Arce-Plaza, A.; Seuret-Jiménez, D.; Morán-Lázaro, J. P.; Sánchez-Rodríguez, F. J. A Theoretical Study on Sb2S3 Solar Cells: The Path to Overcome the Efficiency Barrier of 8%. *Sol. Energy Mater. Sol. Cells* **2019**, *201*, 110123. https://doi.org/10.1016/j.solmat.2019.110123.
(19) Xiao, Y.; Wang, H.; Kuang, H. Numerical Simulation and Performance Optimization of Sb2S3 Solar Cell with a Hole Transport Layer. *Opt. Mater.* **2020**, *108*, 110414. https://doi.org/10.1016/j.optmat.2020.110414.
(20) Chen, C.; Tang, J. Open-Circuit Voltage Loss of Antimony Chalcogenide Solar Cells: Status, Origin, and Possible Solutions. *ACS Energy Lett.* **2020**, *5* (7), 2294–2304. https://doi.org/10.1021/acsenergylett.0c00940.
(21) Wang, S.; Zhao, Y.; Che, B.; Li, C.; Chen, X.; Tang, R.; Gong, J.; Wang, X.; Chen, G.; Chen, T.; Li, J.; Xiao, X. A Novel Multi-Sulfur Source Collaborative Chemical Bath Deposition Technology Enables 8%-Efficiency Sb2S3 Planar Solar Cells. *Adv. Mater.* **2022**, *34* (41), 2206242. https://doi.org/10.1002/adma.202206242.
(22) Liu, X.; Cai, Z.; Wan, L.; Xiao, P.; Che, B.; Yang, J.; Niu, H.; Wang, H.; Zhu, J.; Huang, Y.; Zhu, H.; Zelewski, S. J.; Chen, T.; Hoye, R. L. Z.; Zhou, R. Grain Engineering of Sb_2S_3 Thin Films to Enable Efficient Planar Solar Cells with High Open-Circuit Voltage. *Adv. Mater.* **2023**, 2305841. https://doi.org/10.1002/adma.202305841.
(23) Gao, J.; Tang, R.; Cai, H.; Cai, Z.; Xiao, P.; Li, G.; Jiang, C.; Zhu, C.; Chen, T. Oriented Organization of Poly(3-Hexylthiophene) for Efficient and Stable Antimony Sulfide Solar Cells. *ENERGY Environ. Mater.* **2022**, *n/a* (n/a), e12453. https://doi.org/10.1002/eem2.12453.
(24) Peng, X.; Yang, J.; Zhao, Q.; Gao, H.; Huang, Y.; Wang, H.; Zhu, C.; Tang, R.; Chen, T. Negative-Pressure Sulfurization of Antimony Sulfide Thin Films for Generating a Record Open-Circuit Voltage of 805 mV in Solar Cell Applications. *J. Mater. Chem. A* **2023**, *11* (36), 19298–19307. https://doi.org/10.1039/D3TA04604D.
(25) Tang, R.; Wang, X.; Lian, W.; Huang, J.; Wei, Q.; Huang, M.; Yin, Y.; Jiang, C.; Yang, S.; Xing, G.; Chen, S.; Zhu, C.; Hao, X.; Green, M. A.; Chen, T. Hydrothermal Deposition of Antimony Selenosulfide Thin Films Enables Solar Cells with 10% Efficiency. *Nat. Energy* **2020**, *5* (8), 587–595. https://doi.org/10.1038/s41560-020-0652-3.

(26) Zheng, J.; Liu, C.; Zhang, L.; Chen, Y.; Bao, F.; Liu, J.; Zhu, H.; Shen, K.; Mai, Y. Enhanced Hydrothermal Heterogeneous Deposition with Surfactant Additives for Efficient Sb2S3 Solar Cells. *Chem. Eng. J.* **2022**, *446*, 136474. https://doi.org/10.1016/j.cej.2022.136474.
(27) Lin, W.; Guo, W.-T.; Yao, L.; Li, J.; Lin, L.; Zhang, J.-M.; Chen, S.; Chen, G. Zn(O,S) Buffer Layer for in Situ Hydrothermal Sb2S3 Planar Solar Cells. *ACS Appl. Mater. Interfaces* **2021**, *13* (38), 45726–45735. https://doi.org/10.1021/acsami.1c12501.
(28) Su, M.; Feng, Z.; Feng, Z.; Chen, H.; Liu, X.; Wen, J.; Liu, H. Efficient SnO2/CdS Double Electron Transport Layer for Sb2S3 Film Solar Cell. *J. Alloys Compd.* **2021**, *882*, 160707. https://doi.org/10.1016/j.jallcom.2021.160707.
(29) Qi, Y.; Li, Y.; Lin, Q. Engineering the Charge Extraction and Trap States of Sb2S3 Solar Cells. *Appl. Phys. Lett.* **2022**, *120* (22), 221102. https://doi.org/10.1063/5.0094091.
(30) Barthwal, S.; Singh, S.; Chauhan, A. K.; Prabhu, N. S.; Prabhudessai, A. G.; Ramesh, K. A Comprehensive Insight into Deep-Level Defect Engineering in Antimony Chalcogenide Solar Cells. *Mater. Adv.* **2023**, *4* (23), 5998–6030. https://doi.org/10.1039/D3MA00479A.
(31) Choi, Y. C.; Seok, S. I. Efficient Sb2S3-Sensitized Solar Cells Via Single-Step Deposition of Sb2S3 Using S/Sb-Ratio-Controlled SbCl3-Thiourea Complex Solution. *Adv. Funct. Mater.* **2015**, *25* (19), 2892–2898. https://doi.org/10.1002/adfm.201500296.
(32) Liu, Y.; Li, B.; Liang, X.; Liu, T.; Wang, S.; Li, Z. Reactively Sputtered CdS:O Buffer Layers for Substrate Sb2Se3 Solar Cells. *J. Alloys Compd.* **2023**, *932*, 167313. https://doi.org/10.1016/j.jallcom.2022.167313.
(33) Krautmann, R.; Spalatu, N.; Josepson, R.; Nedzinskas, R.; Kondrotas, R.; Gržibovskis, R.; Vembris, A.; Krunks, M.; Oja Acik, I. Low Processing Temperatures Explored in Sb2S3 Solar Cells by Close-Spaced Sublimation and Analysis of Bulk and Interface Related Defects. *Sol. Energy Mater. Sol. Cells* **2023**, *251*, 112139. https://doi.org/10.1016/j.solmat.2022.112139.
(34) Shim, H.; Kwon, Y. Extraction of Interface Trap Density by Analyzing Organohalide Perovskite and Metal Contacts Using Device Simulation. *AIP Adv.* **2019**, *9* (12), 125203. https://doi.org/10.1063/1.5127959.
(35) Im, S. H.; Lim, C.-S.; Chang, J. A.; Lee, Y. H.; Maiti, N.; Kim, H.-J.; Nazeeruddin, Md. K.; Grätzel, M.; Seok, S. I. Toward Interaction of Sensitizer and Functional Moieties in Hole-Transporting Materials for Efficient Semiconductor-Sensitized Solar Cells. *Nano Lett.* **2011**, *11* (11), 4789–4793. https://doi.org/10.1021/nl2026184.
(36) Zhu, L.; Chen, J.; Liu, R.; Dong, C.; Yang, S.; Chen, T.; Chen, C.; Qiao, Q.; Wang, M. Solution-Processed Compact Sb2S3 Thin Films by a Facile One-Step Deposition Method for Efficient Solar Cells. *Sol. RRL n/a* (n/a), 2100666. https://doi.org/10.1002/solr.202100666.
(37) Deng, M.; Li, S.; Hong, W.; Jiang, Y.; Xu, W.; Shuai, H.; Li, H.; Wang, W.; Hou, H.; Ji, X. Natural Stibnite Ore (Sb2S3) Embedded in Sulfur-Doped Carbon Sheets: Enhanced Electrochemical Properties as Anode for Sodium Ions Storage. *RSC Adv.* **2019**, *9* (27), 15210–15216. https://doi.org/10.1039/C9RA02301A.
(38) Eensalu, J. S.; Mandati, S.; Don, C. H.; Finch, H.; Dhanak, V. R.; Major, J. D.; Grzibovskis, R.; Tamm, A.; Ritslaid, P.; Josepson, R.; Käämbre, T.; Vembris, A.; Spalatu, N.; Krunks, M.; Oja Acik, I. Sb2S3 Thin-Film Solar Cells Fabricated from an Antimony Ethyl Xanthate Based Precursor in Air. *ACS Appl. Mater. Interfaces* **2023**. https://doi.org/10.1021/acsami.3c08547.
(39) Deng, H.; Chen, Z.; Xie, W.; Ishaq, M.; Wu, K.; Feng, X.; Kang, Y.; Wang, W.; Cheng, S. Bulk Defect Passivation for Full-Inorganic Sb2S3 Solar Cells by Sulfur-Atmosphere Recrystallization Process. *Sol. RRL* **2023**, *7* (19), 2300482. https://doi.org/10.1002/solr.202300482.
(40) Mayon, Y. O.; White, T. P.; Wang, R.; Yang, Z.; Catchpole, K. R. Evaporated and Solution Deposited Planar Sb2S3 Solar Cells: A Comparison and Its Significance. *Phys. Status Solidi A* **2016**, *213* (1), 108–113. https://doi.org/10.1002/pssa.201532438.
(41) Zhang, X.; Yoshioka, S.; Loew, N.; Ihara, M. Microstructure Control of Absorber Sb2S3 and P-Type Semiconductor CuSCN for Semiconductor-Sensitized Solar Cells (TiO2/Sb2S3/CuSCN). *ECS Trans.* **2014**, *64* (15), 1. https://doi.org/10.1149/06415.0001ecst.

(42) Muto, T.; Larramona, G.; Dennler, G. Unexpected Performances of Flat Sb2S3-Based Hybrid Extremely Thin Absorber Solar Cells. *Appl. Phys. Express* **2013**, *6* (7), 072301. https://doi.org/10.7567/APEX.6.072301.
(43) Kumar, P.; You, S.; Vomiero, A. CuSCN as a Hole Transport Layer in an Inorganic Solution-Processed Planar Sb2S3 Solar Cell, Enabling Carbon-Based and Semitransparent Photovoltaics. *J. Mater. Chem. C* **2022**, *10* (43), 16273–16282. https://doi.org/10.1039/D2TC03420D.
(44) Aliyar Farhana, M.; Bandara, J. Enhancement of the Photoconversion Efficiency of Sb2S3 Based Solar Cell by Overall Optimization of Electron Transport, Light Harvesting and Hole Transport Layers. *Sol. Energy* **2022**, *247*, 32–40. https://doi.org/10.1016/j.solener.2022.10.025.
